# Lipidomic landscape of circulating extracellular vesicles isolated from adolescents exposed to ethanol intoxication: a sex difference study

**DOI:** 10.1186/s13293-023-00502-1

**Published:** 2023-04-21

**Authors:** Carla Perpiñá-Clérigues, Susana Mellado, José F. Català-Senent, Francesc Ibáñez, Pilar Costa, Miguel Marcos, Consuelo Guerri, Francisco García-García, María Pascual

**Affiliations:** 1 Bioinformatics and Biostatistics Unit, Príncipe Felipe Research Center, C/ Eduardo Primo Yúfera, 3, 46012 Valencia, Spain; 2 Department of Physiology, School of Medicine and Dentistry, University of Valencia, Avda. Blasco Ibáñez, 15, 46010 Valencia, Spain; 3 Department of Molecular and Cellular Pathology of Alcohol, Príncipe Felipe Research Center, 46012 Valencia, Spain; 4 Emergency Department, University Hospital of Salamanca-IBSAL, University of Salamanca, 37007 Salamanca, Spain; 5 Department of Internal Medicine, University Hospital of Salamanca, Institute of Biomedical Research of Salamanca (IBSAL), University of Salamanca, 37007 Salamanca, Spain

**Keywords:** Lipidomics, Extracellular vesicles, Alcohol, Adolescence, Sex-based differences, Functional profiling

## Abstract

**Background:**

Lipids represent essential components of extracellular vesicles (EVs), playing structural and regulatory functions during EV biogenesis, release, targeting, and cell uptake. Importantly, lipidic dysregulation has been linked to several disorders, including metabolic syndrome, inflammation, and neurological dysfunction. Our recent results demonstrated the involvement of plasma EV microRNAs as possible amplifiers and biomarkers of neuroinflammation and brain damage induced by ethanol intoxication during adolescence. Considering the possible role of plasma EV lipids as regulatory molecules and biomarkers, we evaluated how acute ethanol intoxication differentially affected the lipid composition of plasma EVs in male and female adolescents and explored the participation of the immune response.

**Methods:**

Plasma EVs were extracted from humans and wild-type (WT) and Toll-like receptor 4 deficient (TLR4-KO) mice. Preprocessing and exploratory analyses were conducted after the extraction of EV lipids and data acquisition by mass spectrometry. Comparisons between ethanol-intoxicated and control human female and male individuals and ethanol-treated and untreated WT and TLR4-KO female and male mice were used to analyze the differential abundance of lipids. Annotation of lipids into their corresponding classes and a lipid set enrichment analysis were carried out to evaluate biological functions.

**Results:**

We demonstrated, for the first time, that acute ethanol intoxication induced a higher enrichment of distinct plasma EV lipid species in human female adolescents than in males. We observed a higher content of the PA, LPC, unsaturated FA, and FAHFA lipid classes in females, whereas males showed enrichment in PI. These lipid classes participate in the formation, release, and uptake of EVs and the activation of the immune response. Moreover, we observed changes in EV lipid composition between ethanol-treated WT and TLR4-KO mice (e.g., enrichment of glycerophosphoinositols in ethanol-treated WT males), and the sex-based differences in lipid abundance are more notable in WT mice than in TLR4-KO mice. All data and results generated have been made openly available on a web-based platform (http://bioinfo.cipf.es/sal).

**Conclusions:**

Our results suggest that binge ethanol drinking in human female adolescents leads to a higher content of plasma EV lipid species associated with EV biogenesis and the propagation of neuroinflammatory responses than in males. In addition, we discovered greater differences in lipid abundance between sexes in WT mice compared to TLR4-KO mice. Our findings also support the potential use of EV-enriched lipids as biomarkers of ethanol-induced neuroinflammation during adolescence.

**Supplementary Information:**

The online version contains supplementary material available at 10.1186/s13293-023-00502-1.

## Highlights

- Ethanol induces a differential enrichment of plasma EV lipid species in human and murine female adolescents compared to males.
- The function of these lipid species suggests that binge alcohol drinking in human female adolescents could prompt elevated EV biogenesis and a more significant immune response than in males.
- WT mice display more significant disparities in lipid abundance between sexes than TLR4-KO mice.
- The study takes a novel approach—based on bioinformatic analysis of lipidomic data—to studying the sex-based differences in the effect of alcohol.
- Plasma EV lipids represent suitable non-invasive biomarker candidates and could help to explain the mechanisms underlying the neuroinflammatory response after acute intoxication.

**Supplementary Information:**

The online version contains supplementary material available at 10.1186/s13293-023-00502-1.

## Plain language summary

Lipids represent essential components of extracellular vesicles (EVs), playing structural and regulatory functions during EV biogenesis, release, targeting, and cell uptake. Lipidic dysregulation has been linked to several disorders. We evaluated how acute ethanol intoxication differentially affected the lipid composition of plasma EVs in male and female adolescents and explored the participation of the immune response. Plasma EVs were extracted from humans and wild-type (WT) and Toll-like receptor 4 deficient (TLR4-KO) mice. Preprocessing and exploratory analyses were conducted after the extraction of EV lipids and data acquisition by mass spectrometry. Our analysis of differential abundance demonstrated, for the first time, that acute ethanol intoxication induced a higher enrichment of distinct plasma EV lipid species in human female adolescents than in males. We observed a higher content of the PA, LPC, unsaturated FA, and FAHFA lipid classes in females, whereas males showed enrichment in PI. These lipid classes participate in the formation, release, and uptake of EVs and the activation of the immune response. Moreover, we observed changes in EV lipid composition between ethanol-treated WT and TLR4-KO mice (e.g., enrichment of glycerophosphoinositols in ethanol-treated WT males), and the sex-based differences in lipid abundance are more notable in WT mice than in TLR4-KO mice. All data and results generated have been made openly available on a web-based platform (http://bioinfo.cipf.es/sal). Our findings also support the potential use of EV-enriched lipids as biomarkers of ethanol-induced neuroinflammation during adolescence.

**Supplementary Information:**

The online version contains supplementary material available at 10.1186/s13293-023-00502-1.

## Background

Intercellular communication is mediated by direct cell-to-cell contact and the endosomal exocytosis of secreted factors (the secretome) [1]. Extracellular vesicles (EVs), which are secreted by almost every cell type and presented in numerous body fluids, represent an important component of the cell secretome. A range of studies has demonstrated the role of EVs, which contain a wide range of DNA, RNA, lipid, and protein species, in physiological processes and pathological conditions such as inflammation, cancer, and neurodegenerative diseases [2]. While recent research has provided extensive information concerning the protein and micro(mi)RNA content of EVs, we understand less regarding lipids, even though they play critical structural and regulatory roles during EV biogenesis, release, targeting, and cell uptake [3]. EVs often display enrichment in cholesterol, sphingomyelin, and saturated phospholipids, suggesting that EV membranes contain lipid raft-like domains [4–6]. The assembled molecular lipids and membrane-bound proteins determine the structure and function of membrane domains; therefore, a better understanding of the roles of specific proteins and lipids that form EV membranes will provide a wealth of information regarding those mechanisms controlling EV formation, release, and subsequent function. As the specific contents of EVs from different biological fluids contain molecules tightly associated with their cell of origin [6], EVs can be considered a source of non-invasive diagnostic biomarkers for various pathological conditions [7].

As essential structural and functional molecules that impact a range of pathological conditions (including metabolic syndrome, inflammation, and neurological disorders), lipids possess huge diversity in structural and physicochemical properties, which supports their involvement in a wide range of biological functions [7]. Lipidomics has emerged as an innovative discipline that supports the discovery of novel lipid species with relevant biomedical applications [3]. Mass spectrometry-based lipidomics coupled with comprehensive computational strategies for the analysis of the large volume of data generated represents a powerful analytical tool for the identification and quantification of the lipidome of cells, tissues, or bodily fluids, which reveals subtle perturbations caused by, for example, pathological conditions, environmental stressors, or therapeutic agents [8].

Ethanol abuse during adolescence (binge alcohol drinking) can cause neuroinflammation, neurodegeneration, and cognitive dysfunction [9, 10]. Ethanol exposure activates Toll-like receptor 4 (TLR4) in glial cells to induce the release of cytokines and inflammatory mediators, which causes brain damage [11, 12]. Our previous research demonstrated that EVs play a role in the spread of ethanol-induced neuroinflammation by increasing the release of EVs enriched with inflammation‐related proteins and TLR4 response-associated miRNAs [13]. Furthermore, we also discovered that adolescent females display a greater vulnerability than adolescent males to the effects of ethanol since females expressed higher levels of inflammatory molecules (e.g., cytokines, chemokines, and EV microRNAs) than males in plasma [14, 15].

Given the critical roles of EV lipids, we employed a highly sensitive lipidomic strategy to characterize EV lipid species isolated from human and murine plasma, analyze the impact of acute ethanol intoxication on the lipid content of plasma EVs in male and female adolescents, and evaluate the differential functional roles of plasma EV lipids in the activation of immune responses.

## Methods

### Human subjects

Our clinical samples comprised 18 adolescents and young adults (50% females) admitted to the Emergency Department of the University Hospital of Salamanca (Spain) with moderate-to-severe acute ethanol intoxication [14–16]. Acute ethanol intoxication was defined by clinical signs and symptoms (e.g., confusion/disorientation, motor incoordination, unsteady gait, impaired reasoning, and slurred speech), blood alcohol levels (BALs) of > 1 g/L, and consumption of at least five (50 g, males) or four (40 g, females) standard drinks during the six hours before admission. Alcohol intoxication is a clinically harmful condition induced by the ingestion of a large amount of alcohol, which leads to high alcohol levels in the bloodstream [17]. Alcohol intoxication in patients is often defined as a BAL greater than 5.4–17.4 mmol/L (25–80 mg/dL or 0.025–0.080%) [18]. Of note, individuals generally failed to recall the total amount drunk or the time between the first and last intake of ethanol. Exclusion criteria were the presence of other acute (e.g., trauma or infection) or chronic illness, medication use, or suspicion/confirmation of the use of illegal drugs (apart from cannabis). Table 1 describes the clinical, epidemiological, and analytical characteristics of the individuals in this study.

**Table 1:** Characteristics of study individuals displaying acute ethanol intoxication

	Males (*n* = 9)	Females (*n* = 9)
Age (years)	19.67 (0.34)	19.89 (0.50)
BALs (g/L)	2.42 (0.03)	2.12 (0.04)
Aspartate aminotransferase levels (IU/L)	30.33 (2.26)	19.11 (0.42)
Alanine aminotransferase levels (IU/L)	27.22 (3.43)	14.78 (0.49)
Alkaline phosphatase levels (IU/L)	74.22 (3.41)	59.78 (1.22)
*γ*-Glutamyl transpeptidase levels (IU/L)	28.22 (3.55)	12.00 (0.48)
White blood cell count/μL	8738.89 (274.88)	8173.33 (169.82)
Individuals who reported weekend drinking (%)^*^	6 (75.0)	8 (88.89)

Quantitative variables presented as the mean (SEM), and qualitative variables presented as absolute frequencies (percentage). IU, international units. BALs: blood alcohol levels. * A single male individual refused to answer the questionnaire regarding drinking patterns

**Fig. 1:**
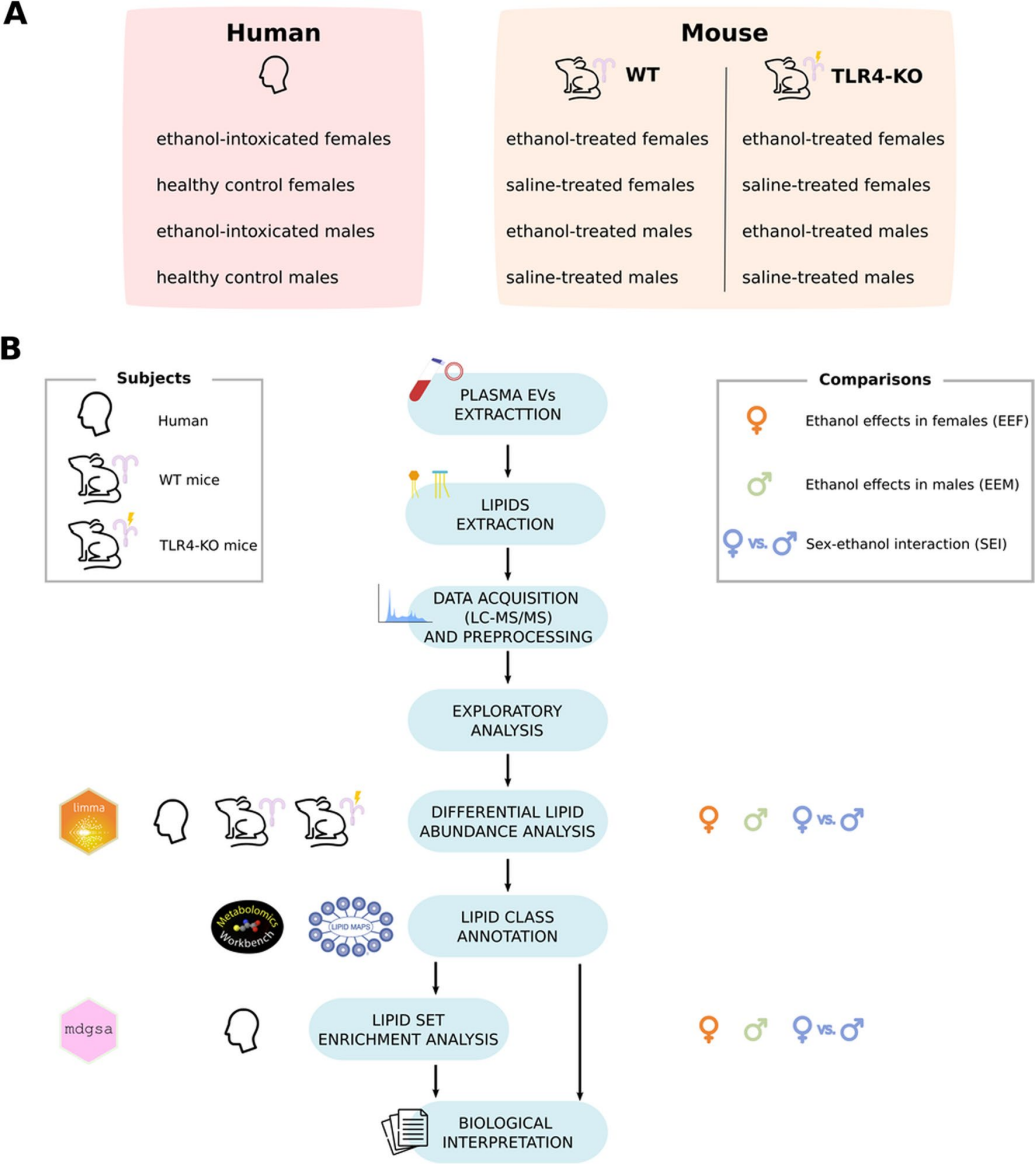
Lipidomic workflow, describing the subjects analyzed and comparisons performed at each step. **A** Human and murine experimental groups. **B** After EV isolation from human and mouse blood plasma, lipids were extracted for quantification and identification by LC–MS/MS. Additional exploratory and differential lipid abundance analyses were also performed. After lipid class annotation, a class enrichment analysis was also carried out for human samples. Finally, functional profiling was applied to interpret the differential abundance analysis results

Eighteen healthy controls (nine males and nine females) recruited from a body of medical and nursing students were also included in the study. Control individuals did not consume alcohol apart from sporadic light drinking, did not report alcohol consumption in the 72 h prior to blood extraction, and did not partake in binge drinking episodes in the three months before the study. These subjects possessed normal hematological and plasma biochemical parameters and did not report any chronic or acute illness. The study was conducted in accordance with the Declaration of Helsinki and was approved by the Ethics Committee of the University Hospital of Salamanca (November 22nd, 2012), and written informed consent was obtained from each participant. Blood samples were obtained from the patients upon admission for standard care and research purposes and used to determine BAL and for complete blood count and liver function evaluations [serum levels of aspartate aminotransferase (IU/L), alanine aminotransferase (IU/L), alkaline phosphatase (IU/L), and γ-glutamyl transpeptidase (IU/L)]. Collected serum was snap-frozen in liquid nitrogen and stored at − 80 °C until further use. Samples were processed and analyzed for this study only after the patients were able to provide informed consent. Figure 1A summarizes the human experimental groups.

### Animals and treatment strategy

C57/BL6 wild-type (WT, *n* = 24) and TLR4-knockout (TLR4-KO, *n* = 24) (C57/BL6 background, kindly provided by Dr. S. Akira, Osaka, Japan) mice were used in this study. Forty-eight animals were used, with six mice per treatment group. Three to four animals were placed in each cage separated by genotype and maintained with water and a solid diet ad libitum under controlled conditions of temperature (23 °C), humidity (60%), and light/dark cycles (12 h/12 h). All experimental procedures were carried out in accordance with the guidelines approved by the European Communities Council Directive (86/609/ECC) and Spanish Royal Decree 53/2013, modified by Spanish Royal Decree 1386/2018 with the approval of the Ethical Committee of Animal Experimentation of the Príncipe Felipe Research Centre (CIPF, Valencia, Spain) on June 19th, 2019 (Project identification code: 2019-08).

To model binge alcohol drinking, morning doses (9–10 a.m.) of saline or 25% (v/v) ethanol (3 g/kg) in isotonic saline were administered intraperitoneally to 30-day-old mice on two consecutive days with 2-day gaps without injections for two weeks (postnatal day [PND] 30 to PND 43), as previously described by Pascual et al. (2007) [19]. Both female and male mice displayed similar or higher BALs of ~ 320 mg⁄dL (peaked at 30 min post-injection) than human ethanol-intoxicated adolescents. Animals were anesthetized 24 h after the last (8th) ethanol or saline administration (PND 44), and whole blood was collected from the hepatic portal vein. After centrifugation, the separated plasma samples were snap-frozen in liquid nitrogen and stored at − 80 °C until use. Figure 1A summarizes the murine experimental groups.

### EV isolation from human and mouse plasma

Plasma EVs were isolated using a total exosome isolation kit (catalog number 4484450, Invitrogen, USA) following the manufacturer's instructions. 250 μL of initial plasma was used to isolate EVs, which were collected and frozen at -80ºC until processing.

### EVs characterization by transmission electron microscopy and nanoparticle tracking analysis

**Fig. 2:**
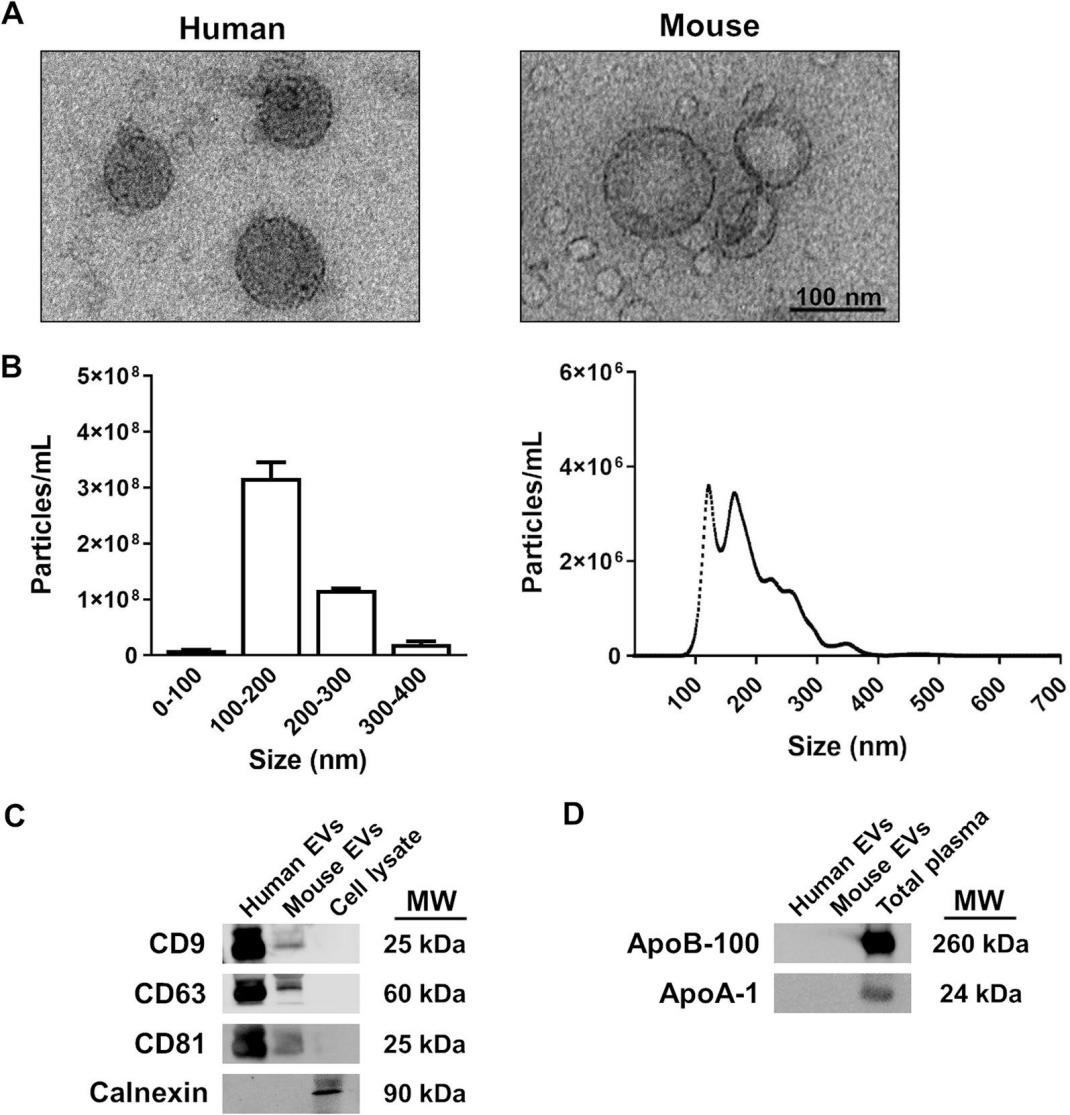
Characterization of plasma EVs. **A** Electron microscopy image of human and murine EVs. **B** Measurement of human EV size distribution and concentration by nanoparticle tracking analysis. A high peak ranging between 100 and 200 nm is shown, which includes the size range of EVs. **C** Analysis of the protein expression of EV markers (CD9, CD63, and CD81) in EVs and cell lysates. Calnexin expression was used to discount cytosolic protein contamination in EV samples. Cell lysates from astroglial cells were used as a positive control for calnexin expression. **D** Expression of ApoB-100 (LDL marker) and ApoA-1 (HDL marker) to measure LDL and HDL contamination in plasma EV samples. Plasma EV samples were not contaminated by LDL and HDL particles. Total plasma was used as a positive control for ApoB-100 and ApoA-1 expression. A representative immunoblot for each protein is shown

Freshly isolated EVs were fixed with 2% paraformaldehyde and prepared as previously described [13]. Preparations were examined under a transmission FEI Tecnai G2 Spirit electron microscope (FEI Europe, Eindhoven, The Netherlands) with a digital camera Morada (Olympus Soft Image Solutions GmbH, Münster, Germany). In addition, the absolute size range and concentration of EVs were analyzed using the NanoSight NS300 Malvern (NanoSight Ltd., Minton Park, UK), as previously described [13]. Figure 2 reports the characterization of EVs by electron microscopy and nanoparticle tracking analysis.

### Western blot analysis

Western blotting was performed in plasma EVs for characterization purposes (Fig. 2) using a protocol described elsewhere [10]. The primary antibodies used were: anti-CD9, anti-CD63, anti-CD81, anti-calnexin (Santa Cruz Biotechnology, USA), anti-ApoB-100, and anti-ApoA-1 (Thermo Fisher Scientific, Illinois, USA). Membranes were washed, incubated with the corresponding HRP-conjugated secondary antibodies, and developed using the ECL system (ECL Plus; Thermo Fisher Scientific). Additional file 1: Figure S1 includes the whole membrane of each protein expression.

### Lipid extraction

Lipids were extracted from equal amounts of plasma EVs (0.2 ml/sample) using a modified Folch extraction procedure. The last phase containing the lipids was transferred to a fresh tube, dry vacuumed with nitrogen, and stored at – 80 °C until further analysis. Dried samples were resuspended with isopropanol for different LC/MS acquisition methods (positive and negative-ion modes).

### LC–MS/MS analysis

In fully automated Q-TOF acquisition mode, a pooled human lipid extract representing the 36 samples (four conditions × nine replicates) was acquired by iterative MS/MS. Detailed experimental methods for chromatography and autoMS/MS mass spectrometry were followed as described before [20, 21] with minor modifications. Briefly, sample separation was performed using an Agilent 1290 Infinity LC system coupled to the 6550 Accurate-Mass QTOF (Agilent Technologies, Santa Clara, CA, USA) with electrospray interface (Jet Stream Technology) operating in positive-ion (3500 V) or negative-ion mode (3000 V) and high sensitivity mode. The optimal conditions for the electrospray interface were a gas temperature of 200 °C, drying gas of 12 L/min, nebulizer of 50 psi, sheath gas temperature of 300 °C, and sheath gas flow of 12 L/min. Lipids were separated on an Infinity Lab Poroshell 120 EC-C18 column (3.0 × 100 mm, 2.7 μm) (Agilent, Santa Clara, CA, USA). Under optimized conditions, the mobile phase consisted of solvent A (10 mM ammonium acetate, 0.2 mM ammonium fluoride in 9:1 water/methanol) and solvent B (10 mM ammonium acetate, 0.2 mM ammonium fluoride in 2:3:5 acetonitrile/methanol/isopropanol) using the following gradient: 0 min 70% B, 1 min 70% B, 3.50 min 86% B, 10 min 86% B, 11 min 100% B, 17 min 100% B operating at 50 °C and a constant flow rate of 0.6 mL/min. The injection volume was 5 µL for positive and negative modes.

Agilent Mass Hunter Workstation Software was employed for the data acquisition. LC/MS Data Acquisition B.10.1 (Build 10.1.48) was operated in auto MS/MS, and the three most intense ions (charge states, 1–2) within a 300–1700 m/z mass range (over a threshold of 5000 counts and 0.001%) were selected for MS/MS analysis. The quadrupole was set to a “narrow” resolution (1.3 m/z), and MS/MS spectra (50–1700 m/z) were acquired until 25,000 total counts or an accumulation time limit of 333 ms. To assure the desired mass accuracy of recorded ions, a continuous internal calibration was performed during analyses using the m/z 121.050873 and m/z 922.009798 signals for positive mode and the m/z 119.03632 and m/z 980.016375 signals for negative mode. Additionally, all-ions MS/MS [22] data were acquired on individual samples, with an MS acquisition rate of three spectra/second and four scan segments 0, 10, 20, and 40 eV.

### Lipid annotator database

Five sets of five iterative MS/MS data files from pooled human cell extracts were analyzed with Lipid Annotator software 1 as the first step in the lipidomic workflow. This study used a novel software tool (Lipid Annotator) [23] with a combination of Bayesian scoring, a probability density algorithm, and non-negative least-squares fit to search a theoretical lipid library (modified LipidBlast) developed by Kind et al. [24, 25] to annotate the MS/MS spectra.

Agilent MassHunter Lipid Annotator Version 1.0 was used for all other data analyses. Default method parameters were used, except only [M + H] + and [M + NH4] + precursors were considered for positive-ion mode analysis, and only [M − H]– and [M + HAc − H]– precursors were considered for negative-ion mode analysis. Agilent MassHunter Personal Compound Database and Library (PCDL) Manager Version B.08 SP1 was used to manage and edit the exported annotations.

### Lipid identification

The lipid Personal Compound Database and Library (PCDL) databases created were used for Batch Targeted Feature Extraction in Agilent Mass Hunter Qualitative version 10.0 on the respective batches of 36 all-ions MS/MS data files. The provided “Profinder—Lipids.m” method was adapted in Mass Hunter Qualitative software with modifications previously described by Sartain et al., 2020 [21]. Data were analyzed using the Find by Formula (FbF) algorithm in MassHunter Qualitative Analysis. This approach uses a modified version of the FbF algorithm, which supports the all-ions MS/MS technique. Mass peaks in the low energy channel are first compared against the PCDL created for compounds with the same m/z values, and then a set of putative identifications is automatically compiled. For this list, the fragment ions in the MS/MS spectra from the PCDL are compared to the ions detected in the high-energy channel to confirm the presence of the correct fragments. The precursors and productions are extracted as ion chromatograms and evaluated using a coelution score. The software calculates a number that accounts for abundance, peak shape (symmetry), peak width, and retention time. The resulting compounds were reviewed in the Mass Hunter Qualitative version; features not qualified were manually removed. Mass Hunter Qualitative results and qualified features were exported as a.cef file.

### Bioinformatic analyses

The strategy applied for this study was based on a transcriptomic analysis workflow. All bioinformatics and statistical analysis were performed using R software v.3.6.3 [26]. Figure 1B illustrates the experimental design.

### Data preprocessing

Data preprocessing included filter entities, normalization of abundance lipid matrix, and exploratory analyses. Mass Hunter Qualitative results (.cef file) were imported into Mass Profiler Professional (MPP) (Agilent Technologies) for statistical analysis, where separate experiments were created for positive and negative-ion modes. Entities were filtered based on frequency, selecting those consistently present in all replicates of at least one treatment. A percentile shift normalization algorithm (75%) was used, and datasets were baselined to the median of all samples. The median of their abundance values was calculated when duplicated lipids with different retention times were present. Data normalization was followed by exploratory analysis using cluster analysis, principal component analysis (PCA), and box and whisker plots by samples and lipids to detect abundance patterns between samples and lipids and batch effects anomalous behavior in the data. At this point, samples behaving in an anomalous manner and outliers (values that lie over 1.5 × interquartile range (IQR) below the first quartile (Q1) or above the third quartile (Q3) in the data set) were excluded for presenting a robust batch effect with a critical impact on differential abundance analysis.

### Differential lipid abundance

Lipid abundance levels between groups were compared using the limma R package [27]. *p*-values were adjusted using the Benjamini and Hochberg (BH) procedure [28], and significant lipids were considered at a BH-adjusted *p*-value of ≤ 0.05.

### Class enrichment analysis

Class annotation was conducted using the *RefMet* database [29] and compared with the *LIPID MAPS* database [30]*.* The classification is hierarchical. As an initial step in this division, lipids were divided into several principal categories (“super classes”) containing distinct main classes and sub classes of molecules, devising a standard manner of representing the chemical structures of individual lipids and their derivatives. Additional file 1: Table S1 and the legends of Figs. 5 and 6 detail all abbreviations. Annotation was followed by ordering the lipids according to the *p*-value and sign of the statistic obtained in the differential lipid abundance. Similar to a Gene Set Enrichment Analysis (GSEA) method, a class enrichment analysis was carried out using Lipid Set Enrichment Analysis (LSEA) implemented in the mdgsa R package [31]. The *p*-values were corrected for BH, and classes with a BH-adjusted *p*-value of ≤ 0.05 were considered significant.

### Comparisons

Three comparisons were performed for each group (human, WT mice, TLR4-KO mice) to analyze differential lipid abundance (Fig. 1B): (i) ethanol effects in females (EEF), which compares ethanol-intoxicated females and control females; (ii) ethanol effects in males (EEM), which compares ethanol-intoxicated males and control males; and (iii) sex–ethanol interaction (SEI), which compares EEF and EEM. Class enrichment analysis was assessed using the same three comparisons in human samples.

The statistics used to measure the differential patterns were the logarithm of fold change (LFC) to quantify the effect of differential lipid abundance analysis and the logarithm of odds ratio (LOR) to measure the enrichment of each functional class. A positive statistical sign indicates a higher mean for the variable in the first element of the comparison, whereas a negative statistical sign indicates a higher mean value for the second element. The SEI comparisons focus on finding differences between female and male comparisons. Thus, a positive statistic may indicate either upregulation in females and downregulation in males or a higher increase or a lower decrease of the variable in intoxicated female subjects. On the other hand, a negative statistic may indicate either upregulation in males and downregulation in females or a higher increase or a lower decrease of the variable in intoxicated male subjects. In this comparison, the behavior of each lipid across the groups must be assessed a posteriori, examining female and male comparisons (Additional file 1: Fig. S2).

In addition, a correlation analysis was conducted between the differential abundance results in the different comparisons, including between humans and mice. Pearson's correlation coefficient measures the relationship between these differential profiles, providing an overall picture, while the intersection of the significant lipids between comparisons provides a specific view of the results of the comparisons. The complementary nature of the approaches improves the understanding of the results of the evaluated comparisons.

### Web platform

All data and results generated in the different steps of the bioinformatics analyses are available on a web platform (http://bioinfo.cipf.es/sal), which is freely accessible to any user and allows the confirmation of the results described in this manuscript. The front-end was developed using the Angular Framework, the interactive graphics used in this web resource have been implemented with plotly [32], and the exploratory analysis cluster plot was generated with the ggplot2 R package [33].

This easy-to-use resource is divided into seven sections: (1) a summary of analysis results; the detailed results of the (2) exploratory analysis and (3) differential abundance for each of the studies; (4) class annotation results; (5) LSEA results, where the user can interact with the web platform through graphics and tables and search for specific information related to lipid species or classes; and (6–7), which include methods, bioinformatics scripts, and Additional file 1.

## Results

### Sex-based differences in the lipid profiles of plasma EVs isolated from human ethanol-intoxicated adolescents

The median age of intoxicated human female and male individuals was 18.0 years (interquartile range (IQR) 18.0–21.0) and 19.0 years (IQR 19.0–20.0), respectively; these ages are considered late adolescence (ages 16–20 years) or young adulthood (ages 21–25 years) [34, 35]. The biochemical analysis of plasma during the intoxication period demonstrated median BALs of 2.10 g/L (IQR 1.80–2.20) for females and 2.40 g/L (IQR 2.25–2.73) for males. We found no evidence of other drugs of abuse in the study subjects. Overall, the BAL data exhibited a broader dispersion in females than males. Control subjects displayed a median age of 21.5 years (IQR 21.0–22.5) for females and 23.0 years (IQR 21.0–23.0) for males.

We analyzed the lipidomic profile of plasma EVs from control and ethanol-intoxicated females and males via LC–MS/MS using negative and positive-ion modes and identified 381 and 276 lipid compounds, respectively. After normalizing sample data, filtering outliers, and summarizing repeated lipids with the median, we obtained 330 and 247 lipids in negative and positive-ion modes.

**Fig. 3:**
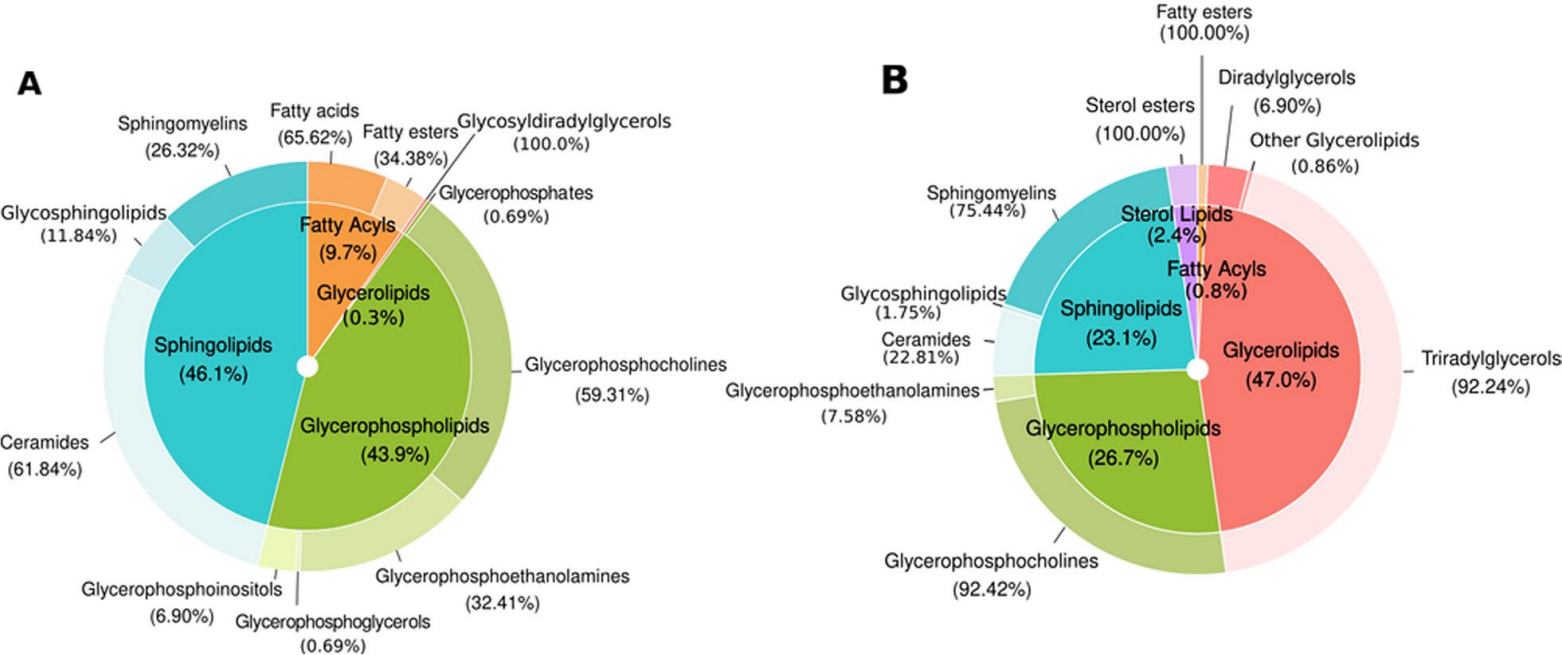
Classification of total lipids in plasma EVs from human adolescents: super class (inner) and main class (outer). The super class percentages display the number of lipids that each super class represents within total lipids. The outer and inner percentages represent the total lipids in super classes and main classes obtained through the negative (**A)** and positive (**B)** ion modes

We used RefMet and LIPID MAPS databases to classify all lipids identified in human subjects to characterize differences in lipid composition between ethanol-intoxicated female and male adolescents at the molecular level. Figure 3 describes the total number of super classes and main classes in the lipid profile from all human plasma EV samples (Additional file 1: Table S2 and S3 include the sub classes). Overall, the EV lipid composition displays enrichment for ceramides, sphingomyelins, glycerophosphocholines, and triradylglycerols, but a lower proportion of Fatty Acids and Fatty Esters.

**Fig. 4:**
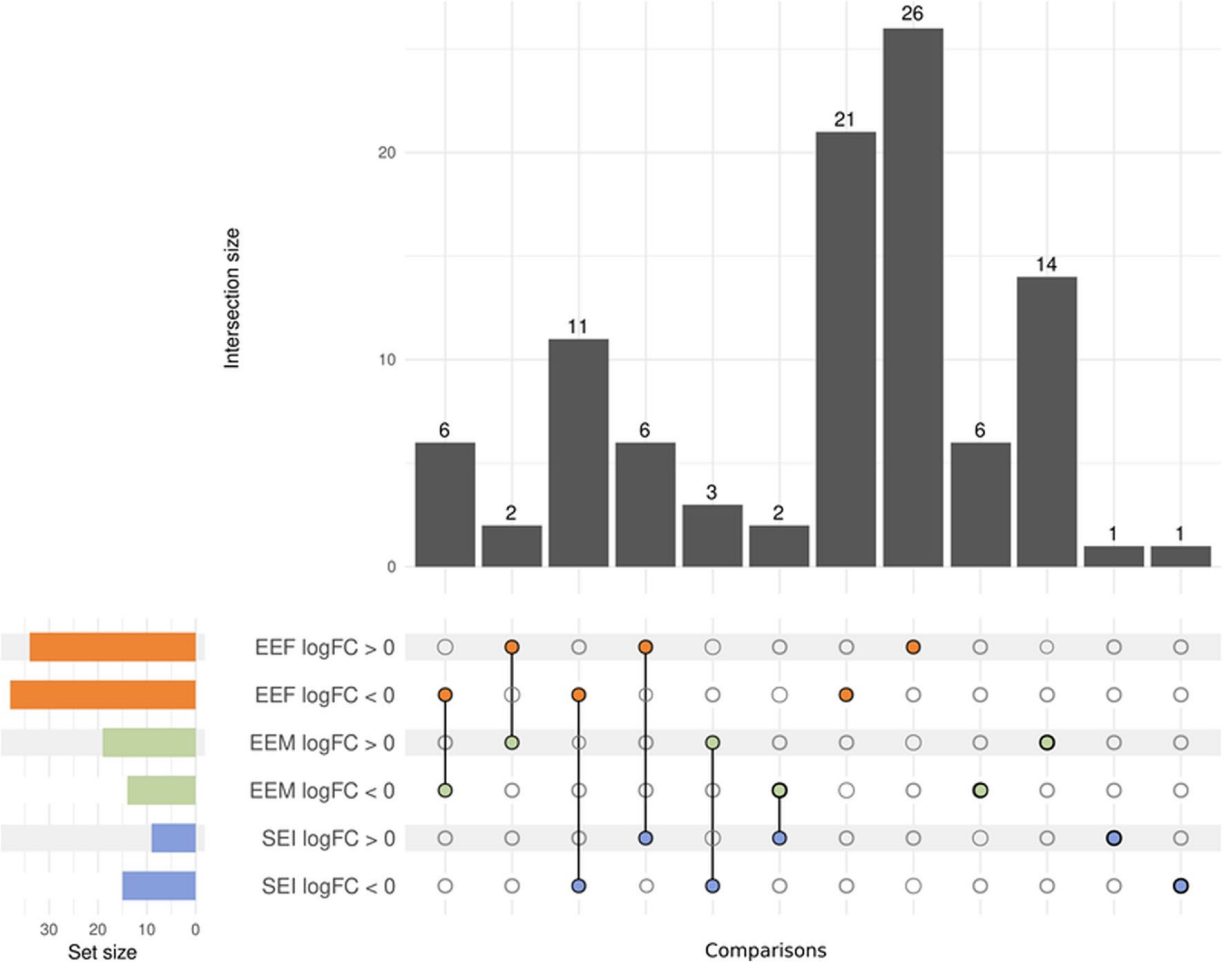
Upset plot of the differential abundance analysis results from human samples. The results of each comparison are separated according to the LFC sign. Horizontal bars indicate the number of significant lipids in each comparison (a specific color for each comparison). Vertical bars indicate the lipids included in the intersection of the groups denoted with a colored dot underneath. A colored dot under a bar indicates the specificity of the genes in this group. Comparisons used: EEF (ethanol effects in females), EEM (ethanol effects in males), and SEI (sex–ethanol interaction)

As described in the Material and Methods section, we employed three comparisons to analyze the differential lipid abundance in human plasma EVs from distinct groups (EEF, ethanol effects in females; EEM, ethanol effects in males; SEI, sex–ethanol interaction). Table 2 describes those lipids that displayed significant changes in abundance in ethanol-intoxicated females and males compared to their respective controls. Ethanol-intoxicated females displayed a significant change in the differential abundance analysis (72 lipids), while ethanol-intoxicated males presented 33 significantly altered lipids. Furthermore, we found eight common lipids in both comparisons in both negative and positive-ion modes (Fig. 4, Additional file 1: Tables S4 and S5). The interaction of both variables (sex and treatment) revealed 24 significant lipid species, with 17 lipid species shared by EEF and SEI comparisons and five species shared by EEM and SEI comparisons (Fig. 4, Additional file 1: Tables S4 and S5).

**Table 2:** Summary of lipids with significant abundance by both ion modes in humans

Ion mode	EEF^1^			EEM^1^			SEI^2^		
	LFC > 0	LFC < 0	Total	LFC > 0	LFC < 0	Total	LFC > 0	LFC < 0	Total
Negative	26	25	51	9	4	13	9	13	22
Positive	8	13	21	10	10	20	0	2	2
Total	34	38	72	19	14	33	9	15	24

Significant lipids separated according to the sign of their log fold-change (LFC). ^**1**^In EEF and EEM comparisons, LFC > 0 columns = differential abundance in lipids increased after ethanol exposure; LFC < 0 columns = differential abundance in lipids decreased after ethanol exposure.^**2**^In SEI, LFC > 0 columns = differential abundance in lipids increased in females; LFC < 0 columns = Differential abundance in lipids increased in males

The relationship between EEF and EEM comparisons of LFC values for all lipids in the differential abundance analysis displayed a positive correlation in both ion modes; however, the correlation coefficient remained close to zero (negative-ion mode 0.22, positive-ion mode 0.30) (Additional file 1: Fig. S3), indicating a lack of any relationship between the variables sex and ethanol intoxication. These results provide robust evidence for sex-based differences in lipid abundance induced by ethanol intoxication.

### Sex-based differences in functional lipid profiling in plasma EVs isolated from human ethanol-intoxicated adolescents

**Fig. 5:**
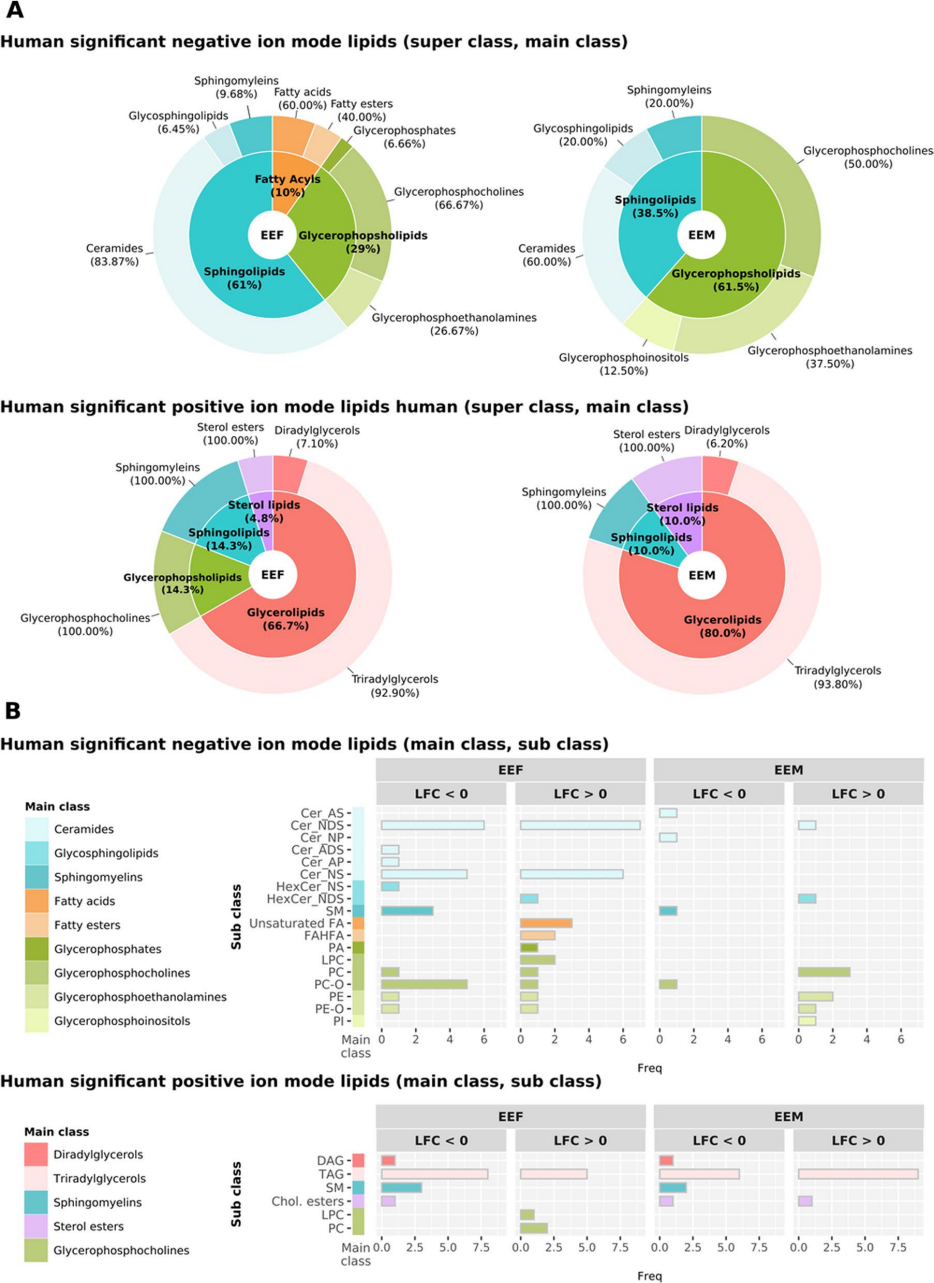
Summary of lipids with significant abundance in humans by class annotation in negative and positive-ion mode. **A** The super class percentages (inner) show the number of lipids each super class represents in the total significant lipids. The main class percentages (outer) show the number of lipids each main class represents in its corresponding super class. **B** Number of significant lipids in the sub classes. The bar charts indicate the number of significant lipids in the sub classes, and the color corresponds to the main class. Comparisons used: EEF (ethanol effects in females), EEM (ethanol effects in males), and SEI (sex–ethanol interaction). Sub-class lipid abbreviations: Cer_AS, ceramide α-hydroxy fatty acid-sphingosine; Cer_NDS, ceramide non-hydroxy fatty acid-dihydrosphingosine; Cer_NP, ceramide non-hydroxy fatty acid-phytosphingosine; Cer_ADS, ceramide α-hydroxy fatty acid-dihydrosphingosine; Cer_AP, ceramide α-hydroxy fatty acid-phytosphingosine; Cer_NS, ceramide non-hydroxy fatty acid-sphingosine; HexCer_NS, glucosylCeramide/HexosylCeramidesnon-hydroxyfatty acid-sphingosine; HexCer_NDS, glucosylCeramide/HexosylCeramidesnon-hydroxyfatty acid-dihydrosphingosine; SM, sphingomyelin; FA, fatty acid; FAHFA, fatty acid ester of hydroxyl fatty acid; PA, phosphatidic acid; LPC, lyso-phosphatidylcholine; PC, phosphatidylcholine; PC-O, etherphosphatidylcholine (EtherPC); PE, phosphatidylethanolamine; PE-O, etherphosphatidylethanolamine (EtherPE); PI, phosphatidylinositol; DAG, diacylglycerol; TAG, triacylglycerol (TG); Chol. esters, cholesterol esters

We classified significant lipid species into functional classes to analyze the lipidomes of plasma EVs in greater depth. Figure 5 reports the distribution of the classifications, which demonstrate significant differences in several of the main classes glycerophosphoinositols, glycerophosphates, glycerophosphocholine, fatty acids, and fatty esters. Sub-class analysis revealed significant enrichment in the PA, LPC, unsaturated FA, and FAHFA in plasma EVs from ethanol-intoxicated female adolescents, whereas only PI displayed upregulation in ethanol-intoxicated males; however, we observed a much lower proportion of the sub-class cholesterol esters in ethanol-intoxicated female adolescents (Fig. 5B).

The lipid species composition of some main classes revealed the highly enriched nature of ceramides in plasma EVs isolated from ethanol-intoxicated and control females; however, we observed the downregulation of some related sub classes in ethanol-intoxicated males (e.g., Cer_AS and Cer_N) and females (e.g., Cer_ADS and Cer_AP). In contrast, other main classes (e.g., diradylglycerols and sphingomyelins) displayed a similar downregulation in abundance in ethanol-intoxicated females and males compared to their respective controls (Fig. 5B).

**Fig. 6:**
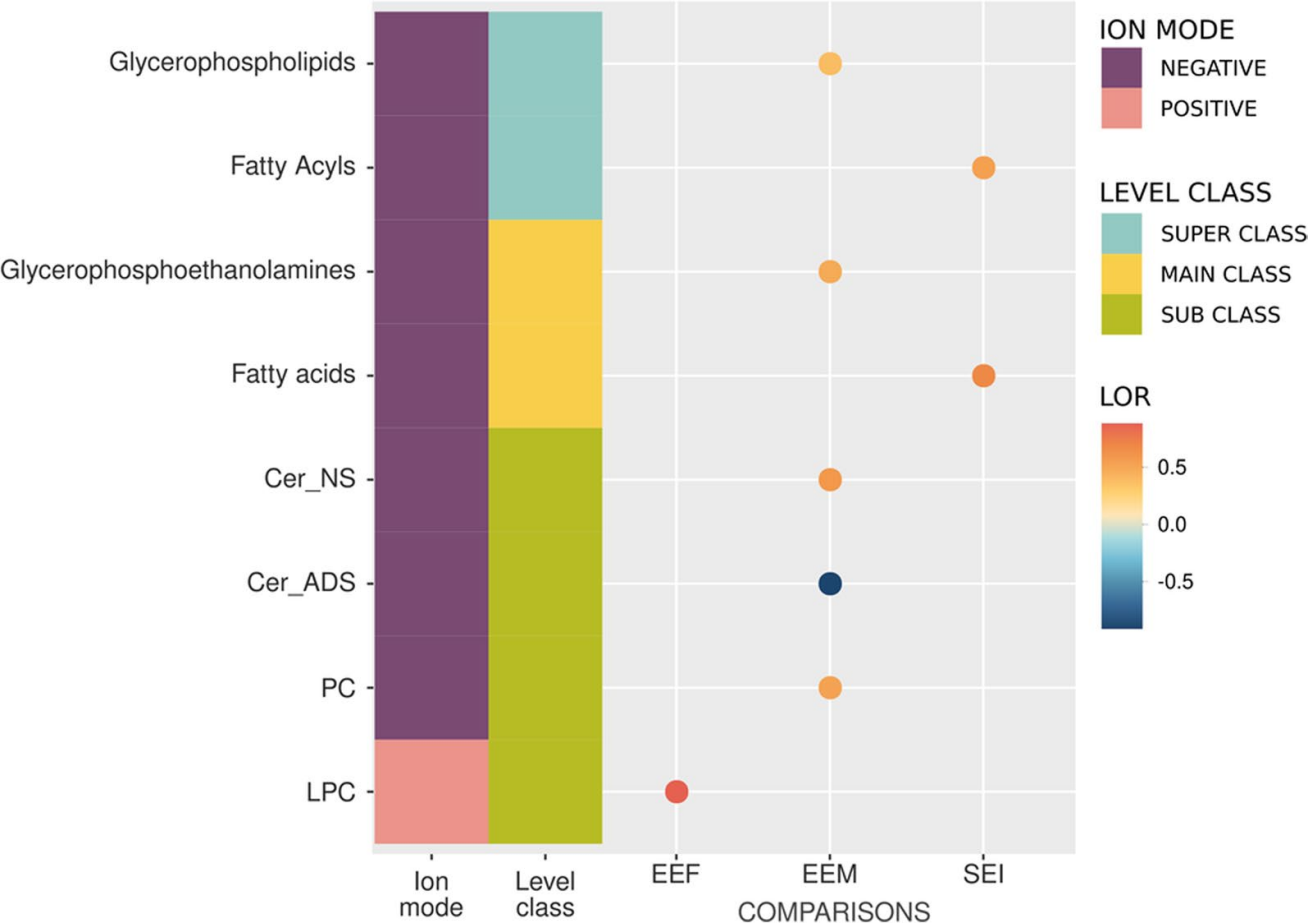
Enriched significant lipid classes in humans by LSEA. The color of the dots represents the sign and magnitude of the change (LOR). Comparisons used: EEF (ethanol effects in females), EEM (ethanol effects in males), and SEI (sex–ethanol interaction). Sub-class lipid abbreviations: Cer_NS, ceramide non-hydroxy fatty acid-sphingosine; Cer_ADS, ceramide α-hydroxy fatty acid-dihydrosphingosine; PC, phosphatidylcholine; LPC, lyso-phosphatidylcholine

The LSEA results of the three human comparisons demonstrated a positive correlation with lipid classes (Fig. 6), which displayed significant differences in abundance (Fig. 5). In addition, we observed a significant enrichment of the fatty acids main class and the fatty acyls super class in the SEI comparison. In EEF, both classes possess a positive LOR value compared to EEM (negative LOR), suggesting higher lipid abundance in ethanol-intoxicated females than males (Fig. 6). We also observed a significantly higher enrichment of the glycerophosphoethanolamines and PC main classes and the Cer_NS sub-class in ethanol-intoxicated males than in control males; however, we also observed a significantly lower enrichment of Cer_ADS in ethanol-intoxicated males. Finally, we encountered a significantly higher enrichment of the LPC sub-class in ethanol-intoxicated females than in control females (Fig. 6).

### Sex-based differences in lipid profiles of plasma EVs isolated from ethanol-treated adolescent mice

We next evaluated potential sex-based differences in ethanol-induced alterations in EV lipid composition and the involvement of the TLR4-mediated immune response in these effects. We analyzed lipid species in control and adolescent ethanol-treated WT and TLR4-KO mice. The lipidomic analyses demonstrated 326 and 289 lipid compounds for the negative and positive-ion modes; we obtained 291 and 264 lipid species in negative and positive-ion modes for further differential analysis after data processing.

**Fig. 7:**
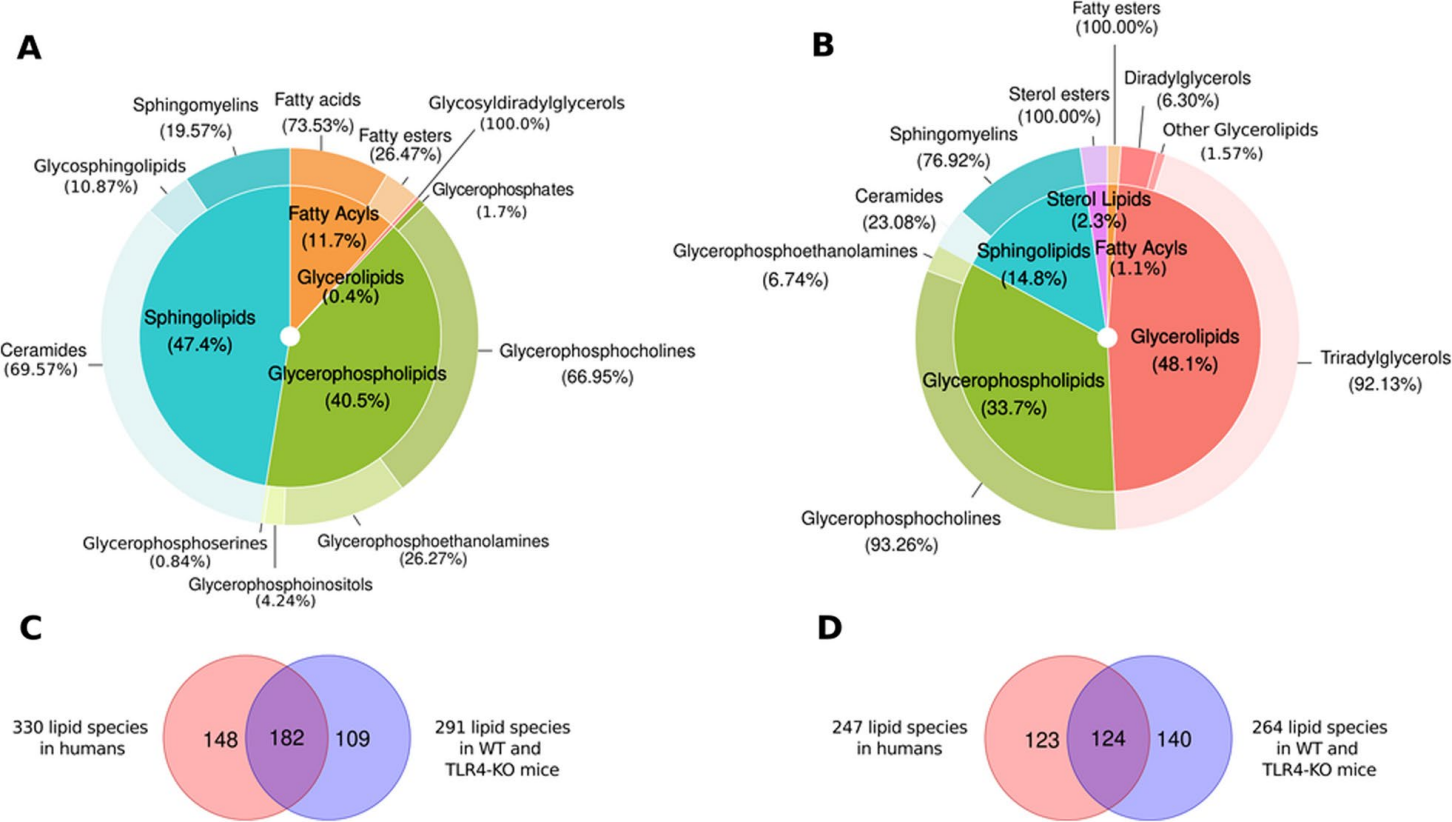
Classification of total lipids in plasma EVs from WT and TLR4-KO adolescent mice: super class (inner) and main class (outer). The super class percentages show the number of lipids that each super class represents in the total lipids. The outer and inner percentages represent the total lipids in the super class and main class obtained through the negative (**A)** and positive (**B)** ion modes. The Venn diagram intersection represents the common lipid species between human and murine samples in negative (**C)** and positive (**D)** ion modes

We employed RefMet and LIPID MAPS databases to characterize the differences in lipid composition between ethanol-treated female and male adolescent mice at the molecular level. The quantitative data analysis obtained for all lipid species in plasma EVs revealed similar percentages of lipid classes (super and main classes, Additional file 1: Tables S6 and S7 include the sub classes) in the negative and positive-ion modes between adolescent mice (Fig. 7A and B) and human individuals (Fig. 3). Figure 7C and D displays the 182 negative and 124 positive common lipids in human and murine samples. Figure 7A and B demonstrates that EV lipid composition in mice displays an enrichment for ceramides, sphingomyelins, glycerophosphocholines, and triradylglycerols with a lower proportion of fatty acids and fatty esters; however, we observed differences in some main and sub classes between human and murine samples (e.g., glycerophosphoglycerol enrichment only occurred in humans, while glycerophosphoserine enrichment only occurred in mice).

**Fig. 8:**
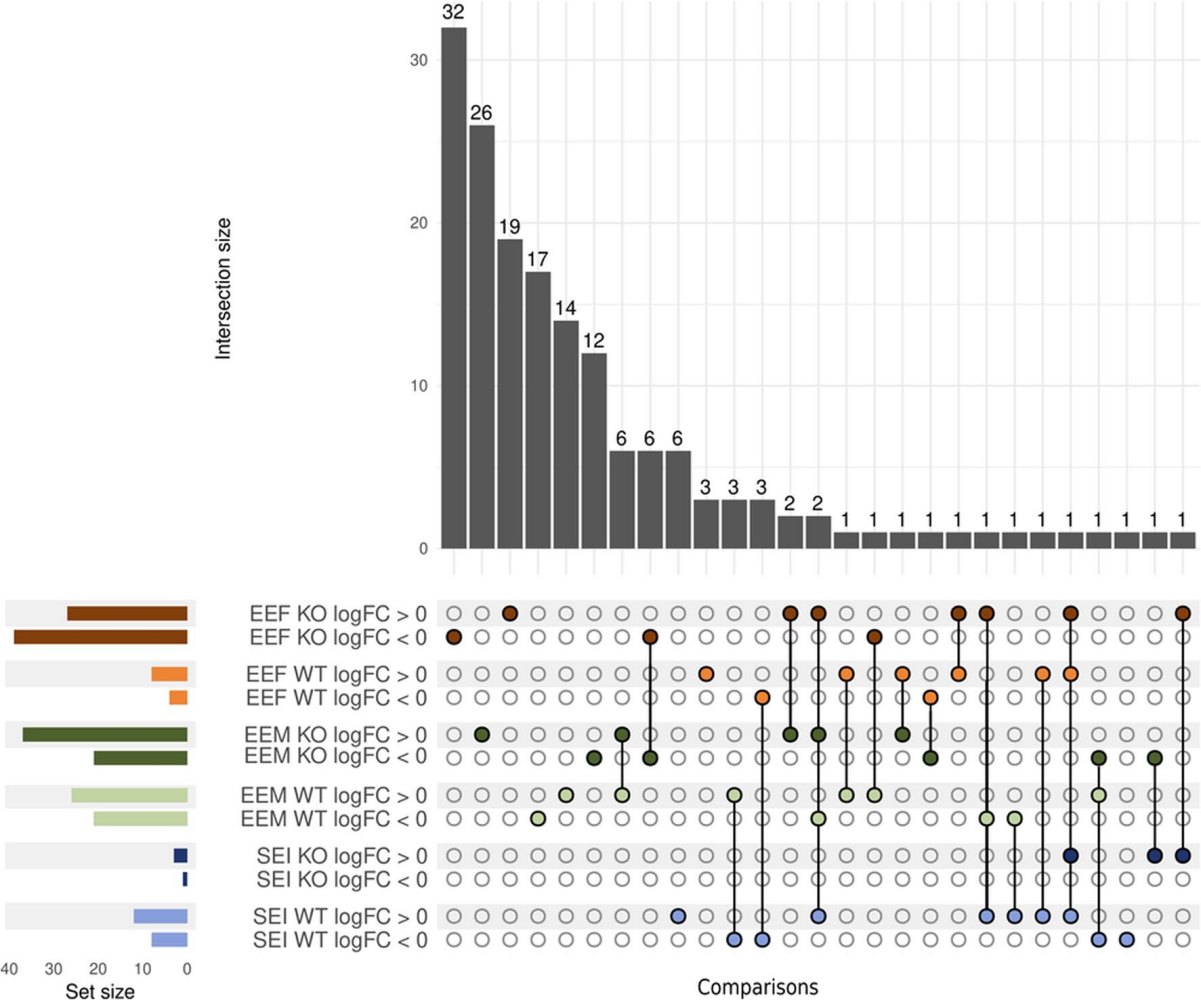
Upset plot of the differential abundance analysis results in WT and TLR4-KO mouse samples. The results of each comparison are separated according to the LFC sign. Horizontal bars indicate the number of significant lipids in each comparison (a specific color for each comparison). Vertical bars indicate the lipids included in the intersection of the groups denoted with a colored dot underneath. A colored dot under a bar indicates the specificity of the genes in this group. Comparisons used in WT and TLR4-KO mice: EEF (ethanol effects in females), EEM (ethanol effects in males), and SEI (sex–ethanol interaction)

Tables 3 and 4 show that ethanol-treated female and male WT mice presented significant changes in 12 and 47 lipid species, respectively; meanwhile, ethanol-treated TLR4-KO female and male mice presented significant changes in 66 and 58 lipid species, respectively. Figure 8 demonstrates that female and male mice treated with ethanol shared one lipid in WT mice and ten lipids in TLR4-KO mice. Female WT and TLR4-KO mice displayed only one significantly differential lipid species in common (Fig. 8); meanwhile, male WT and TLR4-KO mice presented nine common lipid species, although three exhibited different lipid patterns (Fig. 8, Additional file 1: Table S8–S11). In addition, we observed a different lipid abundance pattern between EEF WT and EEM WT (low correlation coefficient) in both lipid ion modes (Additional file 1: Fig. S3); in contrast, we encountered a similar lipid profile for EEF TLR4-KO and EEM TLR4-KO (high and significant correlation coefficient). We also observed a significant positive correlation between human females and WT females (EEF, EEF WT) in lipids (negative-ion mode) (Additional file 1: Fig. S3).

**Table 3:** Summary of lipids with significant abundance using both ion modes in WT mice

Mouse	Ion mode	EEF^1^			EEM^1^			SEI^2^		
		LFC > 0	LFC < 0	Total	LFC > 0	LFC < 0	Total	LFC > 0	LFC < 0	Total
WT	Negative	5	2	7	14	7	21	10	5	15
	Positive	3	2	5	12	14	26	2	3	5
Total		8	4	12	26	21	47	12	8	20

Significant lipids separated according to the sign of their log fold-change (LFC). ^**1**^In EEF and EEM comparisons, LFC > 0 columns = differential abundance in lipids increased after ethanol treatment; LFC < 0 columns = differential abundance in lipids decreased after ethanol treatment.^**2**^In SEI, LFC > 0 columns = differential abundance in lipids increased in females; LFC < 0 columns = Differential abundance in lipids increased in males

**Table 4:** Summary of lipids with significant abundance using both ion modes in TLR4-KO mice

Mouse	Ion mode	EEF^1^			EEM^1^			SEI^2^		
		LFC > 0	LFC < 0	Total	LFC > 0	LFC < 0	Total	LFC > 0	LFC < 0	Total
TLR4-KO	Negative	25	38	63	15	7	22	1	0	1
	Positive	2	1	3	22	14	36	2	0	2
Total		27	39	66	37	21	58	3	0	3

Significant lipids separated according to the sign of their log fold-change (LFC). ^**1**^In EEF and EEM comparisons, LFC > 0 columns = differential abundance in lipids increased after ethanol treatment; LFC < 0 columns = differential abundance in lipids decreased after ethanol treatment.^**2**^In SEI, LFC > 0 columns = differential abundance in lipids increased in females; LFC < 0 columns = differential abundance in lipids increased in males

### Sex-based differences in functional lipid profiling in plasma EVs isolated from ethanol-treated adolescent mice

**Fig. 9:**
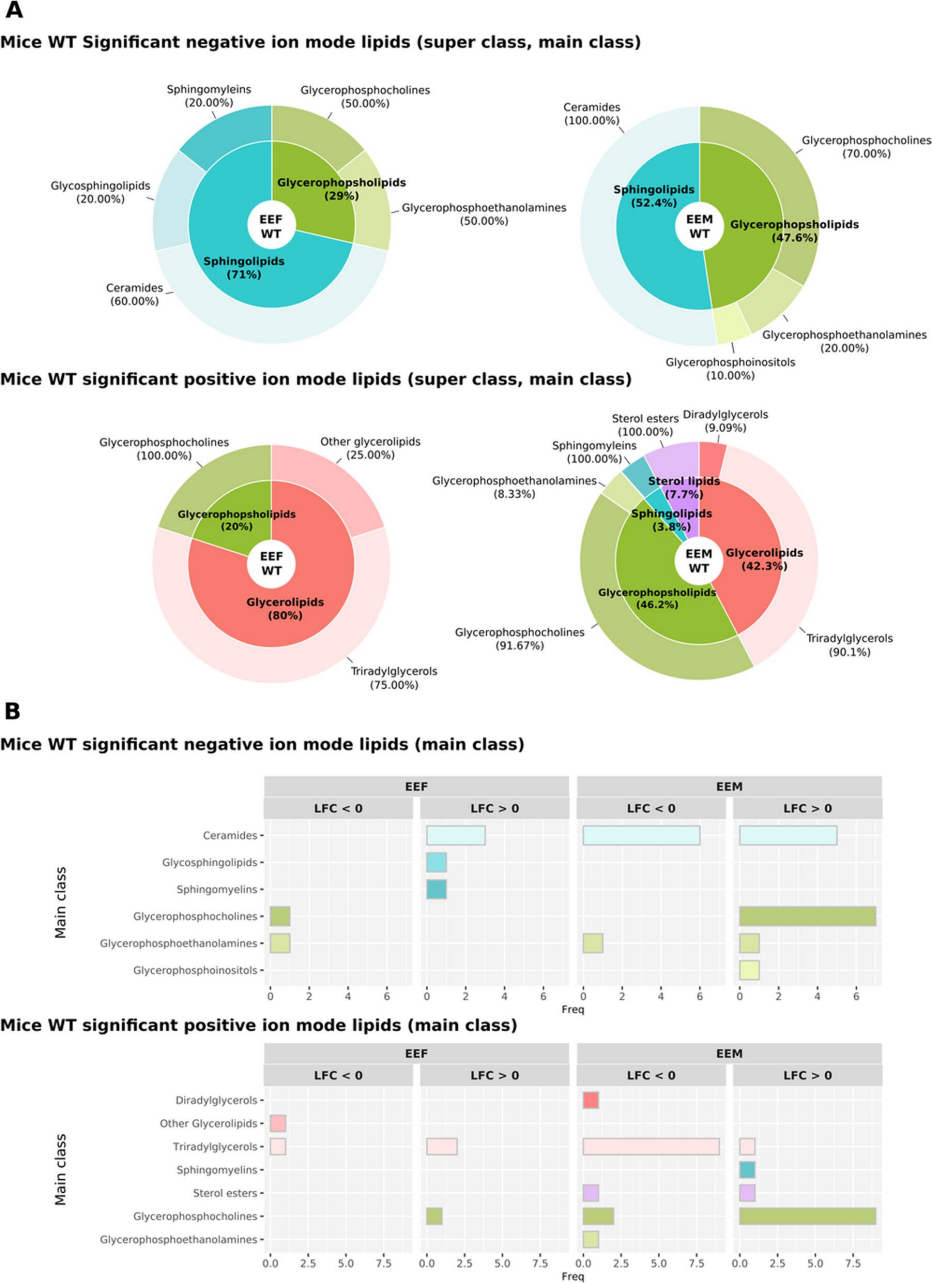
Summary of lipids with significant abundance in WT mice using different class annotations. **A** The super class percentages (inner) show the number of lipids each super class represents in the total significant lipids. The main class percentages (outer) show the number of lipids each main class represents in its corresponding super class. **B** Number of significant lipids in the main classes. The bar charts indicate the number of significant lipids in the main classes. Comparisons used in WT mice: EEF (ethanol effects in females), EEM (ethanol effects in males), and SEI (sex–ethanol interaction)

**Fig. 10:**
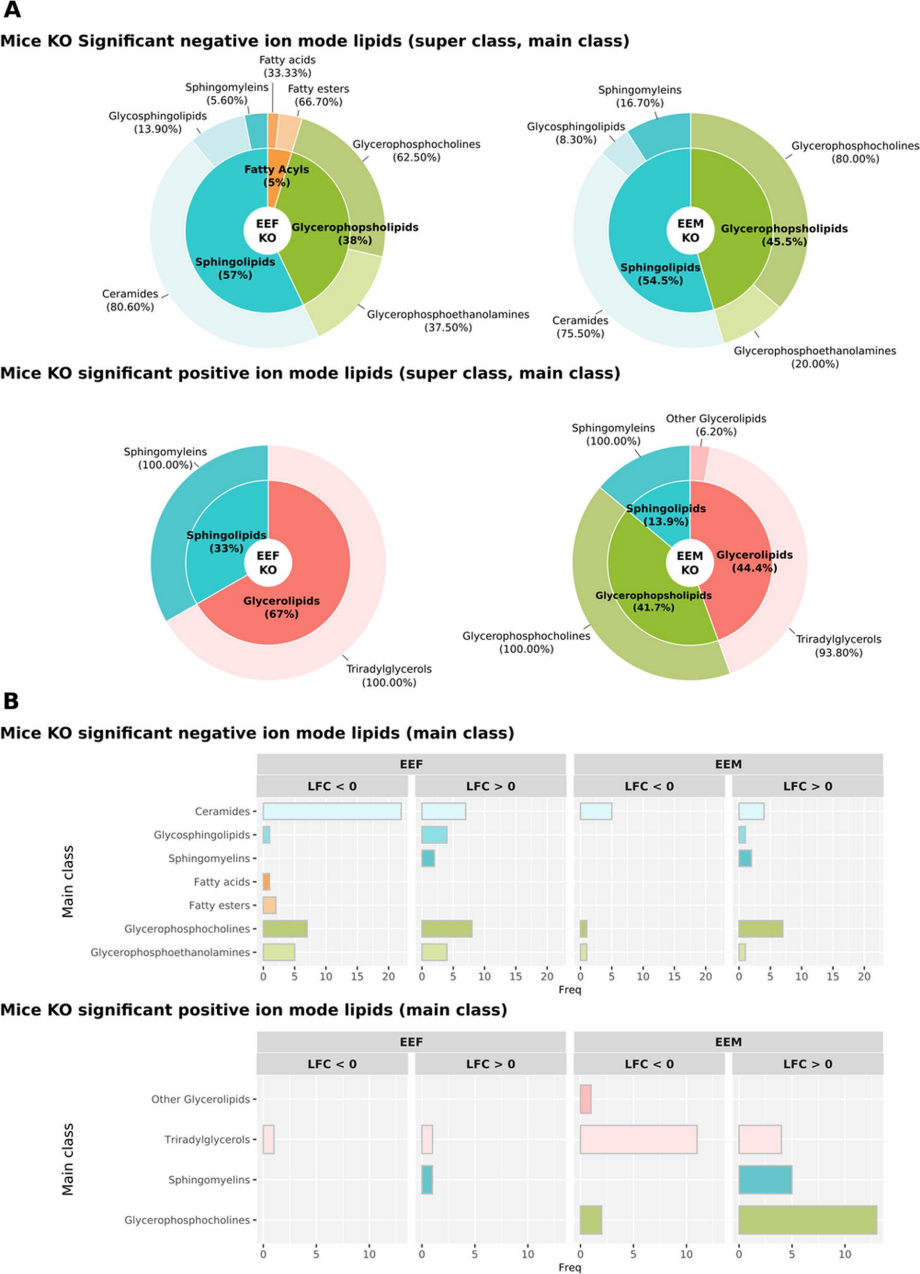
Summary of lipids with significant abundance in TLR4-KO mice using different class annotations. **A** The super class percentages (inner) show the number of lipids each super class represents in the total significant lipids. The main class percentages (outer) show the number of lipids each main class represents in its corresponding super class. **B** Number of significant lipids in the main class. The bar charts indicate the number of significant lipids in the main class. Comparisons used in TLR4-KO mice: EEF (ethanol effects in females), EEM (ethanol effects in males), and SEI (sex–ethanol interaction)

Figure 9 demonstrates the downregulation of most of the main classes of lipids (e.g., glyceroethanolamines and other glycerolipids) in ethanol-treated female WT mice but the upregulation of ceramides, glycosphingolipids and sphingomyelins. Interestingly, we observed the downregulation of Diradylglycerols in ethanol-treated WT males and the upregulation of glycerophosphoinositols and sphingomyelins. When studying alterations in main class lipids in plasma EVs isolated from TLR4-KO mice, we observed differences in the fatty esters and fatty acids main classes in ethanol-treated female TLR4-KO mice and other glycerolipids in ethanol-treated male TLR4-KO mice (Fig. 10). The fatty acyls super class only possessing significance in TLR4-KO mice and the sterol lipids super class only possessing significance in WT animals represent the main differences between ethanol-treated WT and TLR4-KO mice (Figs. 9A, 10A). At the main class level, we observed several significant differences in TLR4-KO and WT mice; however, the results differ in terms of abundance (Figs. 9B, 10B). The other glycerolipids main class became downregulated in ethanol-treated WT females and ethanol-treated TLR4-KO males. In contrast, we only observed the diradylglycerols and glycerophosphoinositols main classes in control and ethanol-treated WT male mice, respectively. When we compared humans and WT mice, only the glycerophosphoinositols main class displayed a common pattern (Figs. 5 and 9).

### Web platform

The web platform (http://bioinfo.cipf.es/sal) contains detailed information regarding the complementary computational approaches involved in this study. This resource includes statistical indicators of each performed analysis for each organism, which users can explore to identify their profiles of interest. This open resource hopes to contribute to data sharing between researchers, elaborating innovative studies, and discovering new findings.

## Discussion

Our recent results demonstrated the involvement of EVs as possible amplifiers and biomarkers of ethanol-induced neuroinflammation. We found an increased level of vulnerability of human female adolescents compared to males to the effects of ethanol, with ethanol-intoxicated females exhibiting fewer anti-inflammatory microRNAs in plasma EVs than males [15]. In addition to microRNAs, EVs also contain various lipid species that could represent regulatory molecules and/or biomarkers. A lipidomic strategy combined with computational data analysis demonstrated, for the first time, that acute ethanol intoxication induces a higher enrichment of EV lipid species (e.g., PA, LPC, unsaturated FA, and FAHFA) in human female adolescents than in males. These lipid species are associated in the formation, release, and uptake of EVs (e.g., PA and LPC) [3, 36–40] and the activation of the immune response (e.g., PA, LPC, and unsaturated FA) [3, 41]. Although we also observed changes in EV lipid composition between ethanol-treated WT and TLR4-KO mice, the sex-based differences in the lipid abundance were more notable in WT mice than in TLR4-KO mice.

Ethanol treatment increases EV release from astroglial cells and enriches their content of inflammation‐related proteins and miRNAs, which may be associated with the amplification of neuroinflammation [13]. Lipid metabolism participates in EV formation and secretion [3, 42], and a recent study revealed that ethanol alters lipid metabolism by increasing cholesterol uptake through mitochondria‐associated endoplasmic reticulum membrane activity, cholesterol esterification, and sphingomyelinase activity in microglia [43]. EVs often display enrichment in cholesterol and sphingomyelin [4], with the conversion of sphingomyelin into ceramide by sphingomyelinases closely linked to EV biogenesis [44]. Accordingly, we observed a decrease in the sphingomyelin sub-class and enrichment of HexCer_NDS and some Cer in plasma EVs from human ethanol-intoxicated females and males, which could be associated with EV formation. In addition, the HexCer_NDS (glycosphingolipids) main class also displayed greater abundance in female ethanol-treated WT mice. This main class participates in EV release, and ceramide may also play a cell-dependent role in EV formation in PC3 cells [45]. Furthermore, ceramides are also essential for the secretion of EVs by facilitating or inducing membrane curvature [3, 36]. Interestingly, ethanol-intoxicated human females (but not males) displayed enrichment in PA and LPC species in plasma EVs. Various reports have described the involvement of the PA sub-class in the formation, secretion, and fusion of EVs and protein–lipid interactions [3, 36–39]. Subra et al. (2010) [40] reported that LPC participated in the fusion of EVs with the endosome-limiting membrane, allowing the release of any EV content into the cytosol.

EVs directly transport lipids from parental cells to recipient cells, which may activate different signaling pathways. For instance, the fatty acids main class or LPC (glycerophosphocholines main class) can induce inflammatory processes or immune responses [3, 46], such as NFκB activation through the TLR4 signaling pathway [41]. In addition, LPS- or phagocytosis-mediated glycerophosphoinositol (e.g., PI) production may participate in the inflammatory responses of macrophages and other immune cells [47]. Herein, we observed that ethanol-intoxicated human female adolescents displayed a significant enrichment of LPC, unsaturated FA, and FAHFA in plasma EVs; however, ethanol-intoxicated males only displayed higher amounts of PI. Overall, these findings suggest that binge drinking in human female adolescents induces a more robust immune response than in males. The enrichment of the noted lipid classes could be used as a biomarker for ethanol-induced neuroinflammation during adolescence since EV lipids already represent robust non-invasive diagnostic and prognostic biomarkers for several brain diseases [48, 49].

TLRs are embedded in cellular membranes, and the posttranslational lipid modification of these membranes regulates the dynamic associations of these receptors with membrane lipid raft microdomains [50]. Our previous studies demonstrated that ethanol induces TLR4 recruitment into lipid rafts upon activation, which leads to the release of cytokines and inflammatory mediators and causes brain damage [11, 12, 51]; furthermore, we revealed that TLR4-deficient mice failed to display an ethanol-induced inflammatory immune response [12]. Recent studies reported that changes in cellular lipid organization might promote or inhibit TLR recruitment into lipid rafts, which can trigger or attenuate receptor-dependent signaling processes [50]. Our results provide evidence for the enrichment of chol. esters and PI in plasma EVs isolated from ethanol-treated WT male mice, whereas no changes occurred in ethanol-treated TLR4-deficient male mice. Cholesterol is required for the biogenesis, release, and stability of EVs and their uptake by target cells [52] and participates in lipid raft formation [5]; meanwhile, as previously mentioned, PI (Glycerophosphoinositols main class) mediates immune responses [47]. In addition, ethanol-treated TLR4-deficient females presented lower levels of unsaturated FA and FAHFA in plasma EVs than in the other comparisons reported in this study. Considering the relation of Fatty acids to inflammation [3, 41], these results suggest that changes in EV lipid composition in ethanol-treated TLR4-deficient mice could inhibit inflammatory immune responses.

We previously demonstrated that both human and WT mouse females displayed a greater level of vulnerability to the effects of ethanol since females expressed higher levels of plasma proinflammatory molecules than males [14, 15]; moreover, a study by Grange et al. revealed similar EV miRNA expression profiles between humans and rat/mouse models [53]. Our current data demonstrate that the Glycerophosphoinositols main class, associated with the immune response [47], becomes upregulated in human and WT mouse males in response to alcohol. The HexCer_NDS (Glycosphingolipids) main class, which has been associated with EV release [45], also increased in human ethanol-intoxicated human adolescents and female ethanol-treated WT mice; however, if we look deeper into the results pertaining to the mouse model of this study, we observe a more notable disparity in lipid abundance between sexes in WT mice than in TLR4-KO mice.

We are aware of certain limitations related to the present study. The use of lipidomic technology in this study represented a challenge concerning the design and application of the bioinformatics strategy to address: (1) the lack of standardization of lipid nomenclature and its integration into the analysis software, (2) the extension of analysis methodologies from genomics and transcriptomics to lipidomics, and (3) the generation of functional annotation [3]. Although there is some evidence that the EVs used in this study might indeed be exosomes, it is actually challenging to distinguish exosomes from microvesicles, as both can be of similar size and express similar markers. Furthermore, we cannot rule out the existence of unknown, significant differences between the human subjects (cases vs. controls) that may limit the comparison regarding lipid composition between groups. Despite these factors, our present study takes a novel approach to the study of sex-based differences in the effect of alcohol based on the bioinformatic analysis of lipidomic data. Our findings will also improve the understanding of the effects of binge drinking by including a gender perspective. All data, results, and programming scripts have been included in open resources (web platform and Zenodo repository) for sharing with the scientific community.

### Perspectives and significance

Our results support the existence of differences between female and male adolescents in EV lipidomic profiles induced in response to binge alcohol drinking. Given the vulnerability of women to alcohol’s effects, this work suggests the use of sex-specific differences in EV lipids to help understand the mechanisms involved during alcohol consumption and provide suitable candidates for non-invasive sex-specific biomarkers (e.g., TG 16:0_18:1_20:3 and Cer_NDS d39:1 in human females and males). The present study takes a novel approach to assess the sex differences in the effect of ethanol on the lipidomic profile through a comprehensive bioinformatic strategy.

## Conclusions

For the first time, these results indicate that ethanol induces a differential enrichment of EV lipid species in human female adolescents compared to males. These lipid species participate in EV formation, release, and uptake, inflammatory immune responses, and TLR4 activation, which suggests that binge alcohol drinking in human female adolescents could be associated with higher levels of EV biogenesis and inflammatory processes than in males. Furthermore, we observed more notable differences in lipid abundance between sexes in WT mice than in TLR4-KO mice. Finally, our sex-based differential analysis of EV-resident lipids could provide suitable candidates for non-invasive biomarkers and help to explain the mechanisms underlying the neuroinflammatory response after acute intoxication. Thus, our approach provides a breakthrough for studies related to one of society's most serious problems, ethanol abuse in the form of binge drinking.

## Supplementary Information

**Additional file 1.** Additional material associated to methods and results.

## Data Availability

The datasets generated and analyzed during the current study and programming scripts are available in the Zenodo repository, http://doi.org/10.5281/zenodo.6581012, and in a web platform: http://bioinfo.cipf.es/sal.
