# Supplementary material for: Lipidomic landscape of circulating extracellular vesicles isolated from adolescents exposed to ethanol intoxication: a sex difference study
Source: Biol Sex Differ. 2023 Apr 21;14:22. doi: 10.1186/s13293-023-00502-1 (PMC10120207; doi:10.1186/s13293-023-00502-1)
Supplement: Supplementary file 1 — Additional file 1. Additional material associated to methods and results. [file 13293_2023_502_MOESM1_ESM.docx]

**Supplementary Material**


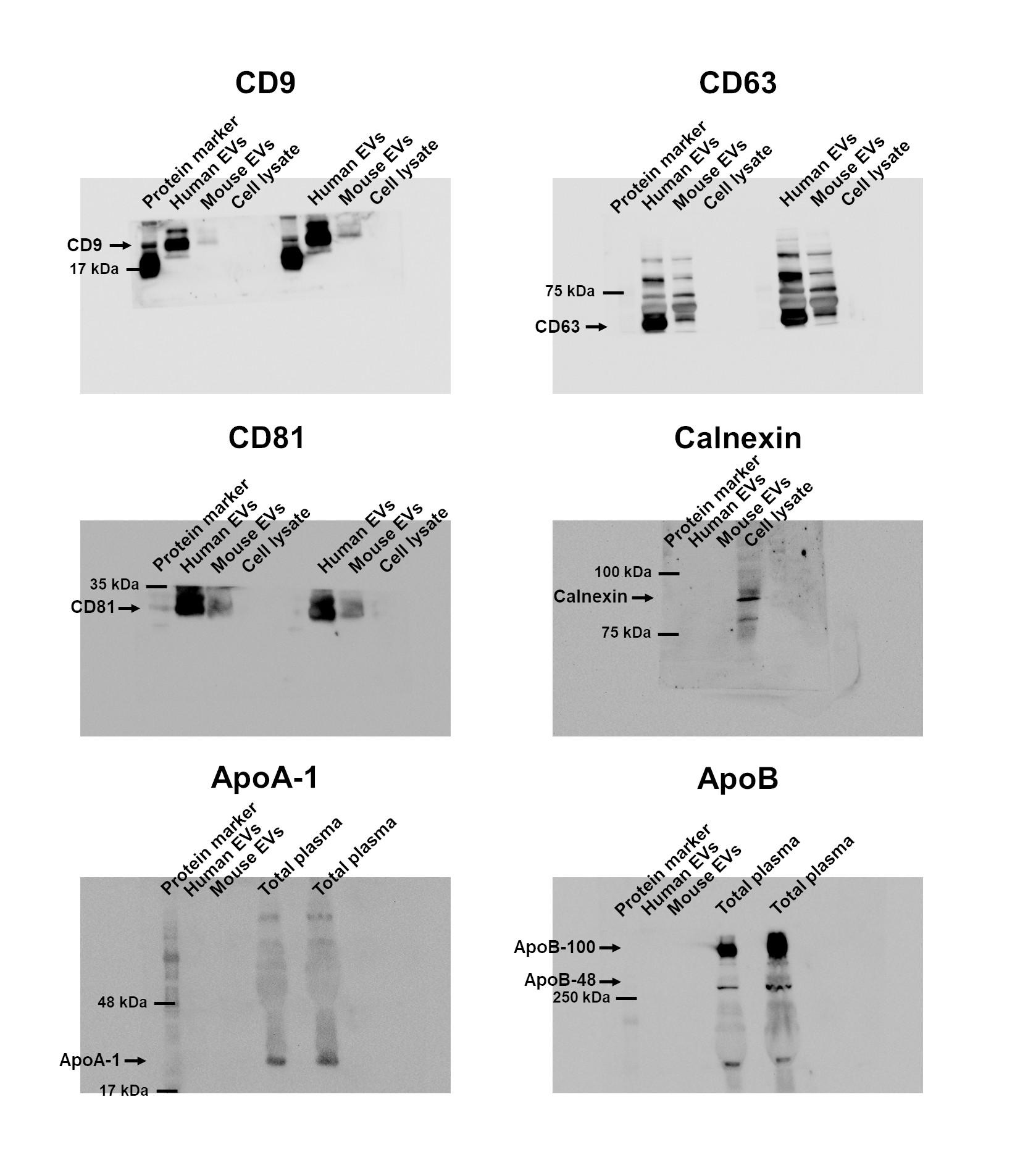
**Figure S1.** Whole blots of CD9, CD63, CD81, calnexin, Apo-1 and ApoB are shown.

**Figure S2.** Bar chart representing the possible causes of a positive (A-E) or negative (F-J) LFC/LOR in the SEI comparison, depending on the EEF or EEM comparison.


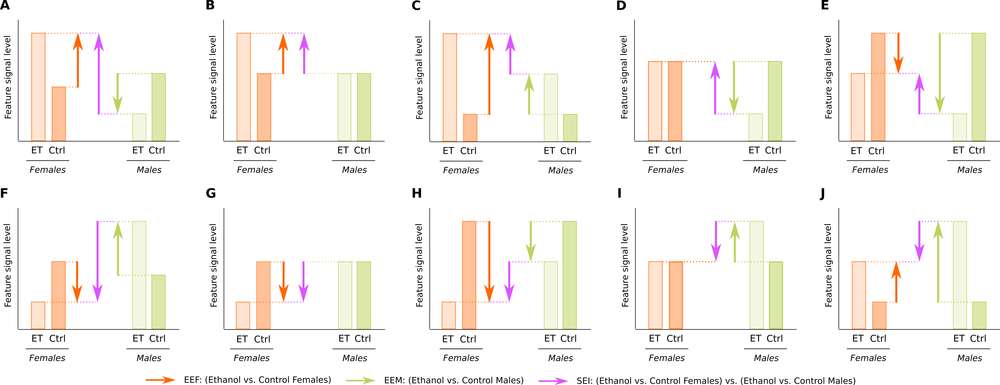


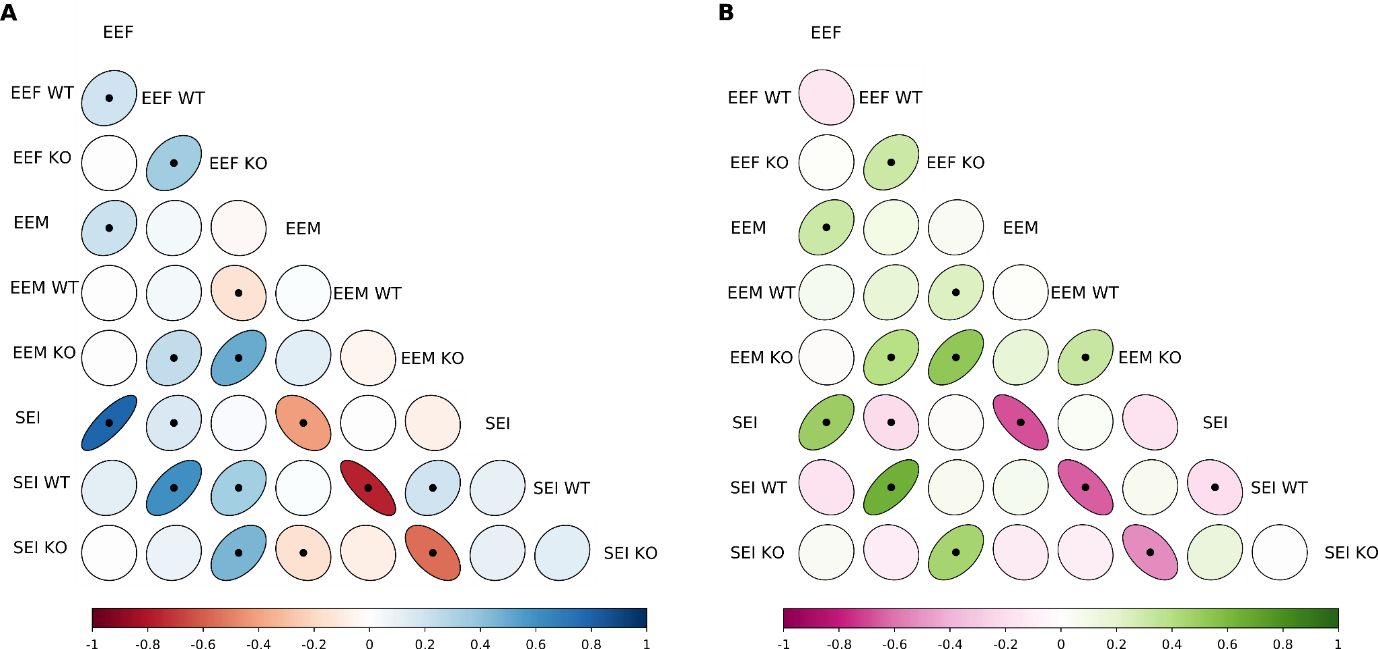
**Figure S3.** Pearson's correlation coefficient in the differential lipid abundance analysis between all the comparisons (EEF: ethanol effects in females; EEM: ethanol effects in males; SEI: sex-ethanol interaction) in all subjects (humans: EEF, EEM, SEI; WT mice: EEF WT, EEM WT, SEI WT; TLR4-KO mice: EEF KO, EEM KO, SEI KO). Plot represents the correlation coefficient and the significant p-value. Black points represent statistically significant correlations. The width of the ellipses and the color gradient indicate the strength of the correlation. A) Negative ion mode lipids, blue and red ellipses represent positive and negative correlations, respectively. B) Positive ion mode lipids, green and pink ellipses represent positive and negative correlations, respectively.

**Table S1.** Abbreviation of the different sub classes.

| CAR | Acylcarnitine |
| --- | --- |
| Cer_ADS | Ceramide α-hydroxy fatty acid-dihydrosphingosine |
| Cer_AP | Ceramide α-hydroxy fatty acid-phytosphingosine |
| Cer_AS | Ceramide α-hydroxy fatty acid-sphingosine |
| Cer_EODS | Ceramide esterified ω-hydroxy fatty acid-dihydrosphingosine |
| Cer_NDS | Ceramide non-hydroxy fatty acid-dihydrosphingosine |
| Cer_NP | Ceramide non-hydroxy fatty acid-phytosphingosine |
| Cer_NS | Ceramide non-hydroxy fatty acid-sphingosine |
| Chol. esters | Cholesterol esters |
| DAG | Diacylglycerol |
| DGTS | Diacylglyceryl-trimethylhomoserine |
| FA | Fatty acid |
| FAHFA | Fatty acid ester of hydroxyl fatty acid |
| HexCer_AP | GlucosylCeramide/HexosylCeramide α-hydroxy fatty acid-phytospingosine |
| HexCer_NDS | GlucosylCeramide/HexosylCeramidesnon-hydroxyfatty acid-dihydrosphingosine |
| HexCer_NS | GlucosylCeramide/HexosylCeramidesnon-hydroxyfatty acid-sphingosine |
| LPC | Lyso-phosphatidylcholine |
| LPE | Lyso-phosphatidylethanolamine |
| OxPC-O | Oxidized etherphosphatidylcholine (OxEtherPC) |
| OxPI | Oxidized Phosphatidylinositols |
| PA | Phosphatidic acid |
| PC | Phosphatidylcholine |
| PC-O | Etherphosphatidylcholine (EtherPC) |
| PE | Phosphatidylethanolamine |
| PE-O | Etherphosphatidylethanolamine (EtherPE) |
| PG | Phosphatidylglycerol |
| PI | Phosphatidylinositol |
| SHexCer | SulfoglucosylCeramide/SulfohexosylCeramide |
| SM | Sphingomyelin |
| SQDG | Sulfoquinovosyl-diacylglycerol |
| TAG | Triacylglycerol (TG) |

**Table S2.** Classification by levels of all negative ion mode lipids in human samples.

| **- NEGATIVE ION MODE IN HUMAN LIPID SAMPLES -** | | | | | |
| --- | --- | --- | --- | --- | --- |
| **SUPER CLASS** | **N** | **MAIN CLASS** | **N** | **SUB CLASS** | **N** |
| Sphingolipids | 152 | Sphingomyelins | 40 | SM | 40 |
|  |  | Glycosphingolipids | 18 | HexCer_AP | 1 |
|  |  |  |  | HexCer_NDS | 11 |
|  |  |  |  | HexCer_NS | 4 |
|  |  |  |  | SHexCer | 2 |
|  |  | Ceramides | 94 | Cer_ADS | 17 |
|  |  |  |  | Cer_AP | 11 |
|  |  |  |  | Cer_AS | 6 |
|  |  |  |  | Cer_EODS | 1 |
|  |  |  |  | Cer_NDS | 35 |
|  |  |  |  | Cer_NP | 2 |
|  |  |  |  | Cer_NS | 22 |
| Fatty Acyls | 32 | Fatty acids | 21 | Unsaturated FA | 21 |
|  |  | Fatty esters | 11 | FAHFA | 11 |
| Glycerophospholipids | 145 | Glycerophosphates | 1 | PA | 1 |
|  |  | Glycerophosphocholines | 86 | PC | 43 |
|  |  |  |  | PC-O | 30 |
|  |  |  |  | LPC | 12 |
|  |  |  |  | OxPC-O | 1 |
|  |  | Glycerophosphoethanolamines | 47 | PE | 27 |
|  |  |  |  | PE-O | 19 |
|  |  |  |  | LPE | 1 |
|  |  | Glycerophosphoinositols | 10 | PI | 9 |
|  |  |  |  | OxPI | 1 |
|  |  | Glycerophosphoglycerols | 1 | PG | 1 |
| Glycerolipids | 1 | Glycosyldiradylglycerols | 1 | SQDG | 1 |
| Total | 330 |  | 330 |  | 330 |

**"**N" represents the total number in each class.

**Table S3.** Classification by levels of all positive ion mode lipids in human samples.

| **- POSITIVE ION MODE IN HUMAN LIPID SAMPLES -** | | | | | |
| --- | --- | --- | --- | --- | --- |
| **SUPER CLASS** | **N** | **MAIN CLASS** | **N** | **SUB CLASS** | **N** |
| Sphingolipids | 57 | Ceramides | 13 | Cer_NS | 13 |
|  |  | Glycosphingolipids | 1 | HexCer_NS | 1 |
|  |  | Sphingomyelins | 43 | SM | 43 |
| Fatty Acyls | 2 | Fatty esters | 2 | CAR | 2 |
| Glycerophospholipids | 66 | Glycerophosphocholines | 61 | PC | 43 |
|  |  |  |  | LPC | 18 |
|  |  | Glycerophosphoethanolamines | 5 | PE | 5 |
| Glycerolipids | 116 | Diradylglycerols | 8 | DAG | 8 |
|  |  | Other Glycerolipids | 1 | SQDG | 1 |
|  |  | Triradylglycerols | 107 | TAG | 107 |
| Sterol Lipids | 6 | Sterol esters | 6 | Chol. esters | 6 |
| Total | 247 |  | 247 |  | 247 |

**"**N" represents the total number in each class.

**Table S4.** Lipids with significant differential abundance in human samples, separated by LFC.

| Negative ion mode | EEF | EEM | SEI |
| --- | --- | --- | --- |
| LFC>0 | Cer_NDS d38:1, **Cer_NDS d39:1**, Cer_NDS d41:2, Cer_NDS d43:1, Cer_NDS d43:2, Cer_NDS d44:1, Cer_NDS d44:2, Cer_NS d18:1_16:0, Cer_NS d18:1_18:0, Cer_NS d18:1_22:0, Cer_NS d18:1_24:0, Cer_NS d18:2_16:0, Cer_NS d18:2_25:0, EtherPC 16:0e_20:3, EtherPE 16:1e_22:6, FA 18:2, FA 19:1, FA 22:4, FAHFA 16:0_12:0, FAHFA 18:2_18:1, HexCer_NDS d42:2, LPC 20:0/0:0, LPC 20:2/0:0, PA 2:0_16:3, PC 14:0_22:6, PE 20:0_20:4 | **Cer_NDS d39:1**, EtherPE 18:1e_22:4, HexCer_NDS d43:1, PC 14:0_18:2, PC 16:0_16:0, *PC 16:0_16:1*, PE 18:0_18:1, PE 36:3, PI 34:1 | Cer_AP t42:1, *Cer_AS d43:1*, Cer_NDS d38:1, Cer_NDS d41:2, Cer_NDS d43:2, Cer_NDS d44:1, Cer_NDS d44:2, FA 19:1, *SM d44:3* |
| LFC<0 | Cer_ADS d42:0, Cer_AP t45:0, Cer_NDS d25:0_18:2, Cer_NDS d36:1, Cer_NDS d40:0, Cer_NDS d40:1, Cer_NDS d42:0, Cer_NDS d42:1, Cer_NS d16:1_22:0, Cer_NS d16:1_23:0, Cer_NS d18:1_25:0, Cer_NS d18:2_23:0, Cer_NS d44:1, EtherPC 16:0e_14:0, **EtherPC 18:0e_22:6**, EtherPC 18:1e_22:6, EtherPC 34:1e, EtherPC 44:4e, EtherPE 18:2e_18:1, HexCer_NS d20:2_22:0, PC 19:0_18:2, PE 14:0_16:0, SM d18:0_14:0, SM d33:2, SM d38:0 | *Cer_AS d43:1*, Cer_NP t43:1, **EtherPC 18:0e_22:6**, *SM d44:3* | Cer_NDS d36:1, Cer_NDS d42:0, Cer_NS d16:1_22:0, Cer_NS d16:1_23:0, Cer_NS d18:2_23:0, Cer_NS d44:1, EtherPC 44:4e, EtherPE 18:2e_18:1, *PC 16:0_16:1*, PE 16:0_18:1, SM d18:0_14:0, SM d33:2, SM d38:0 |

Lipids altered in the EEF and EEM comparisons are indicated in boldface, lipids altered in the EEF and SEI comparisons are underlined, and lipids altered in the EEM and SEI comparisons are indicated in purple italics.

**Table S5.** Lipids with significant differential abundance in human samples, separated by LFC.

| Positive ion mode | EEF | EEM | SEI |
| --- | --- | --- | --- |
| LFC>0 | LPC 20:3/0:0, PC 14:0_18:2, PC 35:3, TG 14:0_16:0_20:4, TG 15:0_16:0_18:2, TG 16:0_18:1_18:1, **TG 16:0_18:1_20:3**, TG 56:6 | CE 18:0, *TG 12:0_15:0_16:0*, TG 12:0_16:0_18:2, TG 14:0_15:0_18:2, TG 16:0_17:0_18:0, **TG 16:0_18:1_20:3**, TG 17:0_18:0_18:0, TG 18:1_18:1_22:0, TG 44:1, *TG 53:2* |  |
| LFC<0 | **CE 20:4**, **DG 18:1_18:2**, SM d38:1, SM d42:0, SM d44:1, TG 16:0_16:0_16:0, TG 16:0_18:1_22:0, TG 16:0_18:1_22:4, TG 17:1_18:0_18:2, **TG 49:4**, **TG 51:3**, **TG 52:7**, TG 53:0 | **CE 20:4**, **DG 18:1_18:2**, SM d33:0, SM d36:3, TG 17:1_18:1_18:2, TG 18:0_18:1_18:1, **TG 49:4**, **TG 51:3**, TG 51:4, **TG 52:7** | *TG 12:0_15:0_16:0*, *TG 53:2* |

Lipids altered in the EEF and EEM comparisons are indicated in boldface, lipids altered in the EEF and SEI comparisons are underlined, and lipids altered in the EEM and SEI comparisons are indicated in purple italics.

**Table S6.** Classification by levels of all negative ion mode lipids in WT and KO mouse samples.

| **- NEGATIVE ION MODE IN WT AND TLR4-KO MICE SAMPLES -** | | | | | |
| --- | --- | --- | --- | --- | --- |
| **SUPER CLASS** | **N** | **MAIN CLASS** | **N** | **SUB CLASS** | **N** |
| Sphingolipids | 138 | Sphingomyelins | 27 | SM | 27 |
|  |  | Glycosphingolipids | 15 | HexCer_AP | 2 |
|  |  |  |  | HexCer_NDS | 10 |
|  |  |  |  | HexCer_NS | 3 |
|  |  | Ceramides | 96 | Cer_ADS | 17 |
|  |  |  |  | Cer_AP | 15 |
|  |  |  |  | Cer_AS | 11 |
|  |  |  |  | Cer_EODS | 3 |
|  |  |  |  | Cer_EOS | 1 |
|  |  |  |  | Cer_NDS | 32 |
|  |  |  |  | Cer_NP | 3 |
|  |  |  |  | Cer_NS | 14 |
| Fatty Acyls | 34 | Fatty acids | 25 | Unsaturated FA | 25 |
|  |  | Fatty esters | 9 | FAHFA | 9 |
| Glycerophospholipids | 118 | Glycerophosphates | 2 | PA | 2 |
|  |  | Glycerophosphocholines | 79 | PC | 46 |
|  |  |  |  | PC-O | 15 |
|  |  |  |  | LPC | 16 |
|  |  |  |  | OxPC-O | 2 |
|  |  | Glycerophosphoethanolamines | 31 | PE | 21 |
|  |  |  |  | PE-O | 7 |
|  |  |  |  | LPE | 3 |
|  |  | Glycerophosphoinositols | 5 | PI | 5 |
|  |  | Glycerophosphoserines | 1 | PS | 1 |
| Glycerolipids | 1 | Glycosyldiradylglycerols | 1 | SQDG | 1 |
| Total | 291 |  | 291 |  | 291 |

"N" represents the total number in each class.

**Table S7.** Classification by levels of all positive ion mode lipids in WT and KO mouse samples.

| **- POSITIVE ION MODE IN WT AND TLR4-KO MICE SAMPLES -** | | | | | |
| --- | --- | --- | --- | --- | --- |
| **SUPER CLASS** | **N** | **MAIN CLASS** | **N** | **SUB CLASS** | **N** |
| Sphingolipids | 39 | Ceramides | 7 | Cer_NS | 7 |
|  |  | Glycosphingolipids | 2 | HexCer_NS | 2 |
|  |  | Sphingomyelins | 30 | SM | 30 |
| Fatty Acyls | 3 | Fatty esters | 3 | CAR | 3 |
| Glycerophospholipids | 89 | Glycerophosphocholines | 83 | PC | 49 |
|  |  |  |  | LPC | 34 |
|  |  | Glycerophosphoethanolamines | 6 | PE | 6 |
| Glycerolipids | 127 | Diradylglycerols | 8 | DAG | 8 |
|  |  | Other Glycerolipids | 2 | DGTS | 2 |
|  |  | Triradylglycerols | 117 | TAG | 117 |
| Sterol Lipids | 6 | Sterol esters | 6 | Chol. esters | 6 |
| Total | 264 |  | 264 |  | 264 |

"N" represents the total number in each class.

**Table S8.** Lipids with significant differential abundance in WT mouse samples, separated by LFC.

| Negative ion mode  **WT mice** | EEF | EEM | SEI |
| --- | --- | --- | --- |
| LFC>0 | Cer_AS d27:1_16:0, Cer_AS d41:1, Cer_AS d42:1, HexCer_NS d40:2, SM d35:1 | Cer_AS d42:1, *Cer_NDS d40:1*, Cer_NS d18:1_16:0, Cer_NS d18:1_23:0, Cer_NS d18:1_24:0, EtherPC 16:0e_18:2, LPC 20:3/0:0, PC 16:0_17:1, PC 18:0_18:2, PC 18:0_22:6, PC 18:2_20:4, PC 20:4_22:6, PE 40:2, *PI 40:5* | Cer_ADS d19:0_15:0, Cer_ADS d38:0, Cer_ADS d41:0, Cer_AS d27:1_16:0, Cer_AS d41:1, *Cer_NDS d41:1*, *Cer_NDS d42:1*, *Cer_NS d18:1_22:0*, *Cer_NS d39:1*, EtherPC 24:0e |
| LFC<0 | PC 18:1_22:6, PE 18:0_22:5 | *Cer_NDS d41:1*, *Cer_NDS d42:1*, Cer_NS d18:1_18:0, *Cer_NS d18:1_22:0*, *Cer_NS d39:1*, Cer_NS d42:3, PE 18:0_22:6 | *Cer_NDS d40:1*, PC 16:1_20:4, PC 18:1_22:6, PE 18:0_22:5, *PI 40:5* |

Lipids altered in the EEF and EEM comparisons are indicated in boldface, lipids altered in the EEF and SEI comparisons are underlined, and lipids altered in the EEM and SEI comparisons are indicated in purple italics.

**Table S9.** Lipids with significant differential abundance in WT mouse samples, separated by LFC.

| Positive ion mode  **WT mice** | EEF | EEM | SEI |
| --- | --- | --- | --- |
| LFC>0 | PC 18:0_22:6, TG 15:0_18:2_18:3, TG 18:2_18:2_21:0 | CE 18:1, LPC 0:0/18:0, LPC 17:0/0:0, *LPC 18:1/0:0*, LPC 20:3/0:0, LPC 22:3/0:0, LPC 22:5/0:0, PC 40:5, *PC 40:8*, PC 44:4, SM d43:2, TG 18:0_18:1_20:3 | TG 49:4, TG 54:1 |
| LFC<0 | DGTS 38:2, TG 53:6 | CE 20:5, DG 18:1_18:2, PC 16:0_20:4, PC 32:2, PE 16:0_18:2, TG 12:0_16:1_16:1, TG 14:0_16:1_18:2, TG 15:0_18:2_22:6, TG 16:0_18:2_22:6, TG 16:1_18:2_18:3, TG 18:0_20:1_20:4, TG 18:1_18:1_22:5, TG 18:1_20:4_22:6, TG 50:6 | *LPC 18:1/0:0*, *PC 40:8*, TG 53:6 |

Lipids altered in the EEF and EEM comparisons are indicated in boldface, lipids altered in the EEF and SEI comparisons are underlined, and lipids altered in the EEM and SEI comparisons are indicated in purple italics.

**Table S10.** Lipids with significant differential abundance in TLR4-KO mouse samples, separated by LFC.

| Negative ion mode **TLR4-KO mice** | EEF | EEM | SEI |
| --- | --- | --- | --- |
| LFC>0 | Cer_AS d27:1_16:0, Cer_NDS d16:0_28:2, Cer_NDS d34:1, **Cer_NDS d42:1**, Cer_NDS d42:2, **Cer_NS d18:1_22:0**, Cer_NS d39:1, EtherPC 16:1e_18:2, EtherPC 18:0e_20:4, EtherPC 18:1e_22:6, EtherPE 16:1e_20:4, EtherPE 16:1e_22:6, HexCer_NDS d24:0_18:1, HexCer_NDS d34:1, HexCer_NDS d42:2, HexCer_NS d20:2_22:1, LPC 20:0/0:0, LPC 20:2/0:0, LPC 22:0/0:0, PC 18:0_22:4, PC 38:2, PE 16:0_18:0, **PE 20:0_20:3**, SM d35:1, **SM d38:1** | Cer_ADS d40:0, Cer_NDS d41:2, **Cer_NDS d42:1**, **Cer_NS d18:1_22:0**, EtherPC 16:0e_18:2, HexCer_AP t18:0_16:0, LPC 18:0/0:0, LPC 19:0/0:0, LPC 20:3/0:0, PC 17:0_18:2, PC 18:0_22:6, PC 19:0_20:4, **PE 20:0_20:3**, **SM d38:1**, SM d41:1 | Cer_AS d27:1_16:0 |
| LFC<0 | Cer_ADS d44:0, Cer_ADS d45:0, Cer_ADS d46:0, Cer_ADS d46:1, **Cer_ADS d47:0**, Cer_ADS d48:0, Cer_AP t22:0_25:0, Cer_AP t41:0, Cer_AP t42:1, Cer_AP t46:1, Cer_AS d44:1, **Cer_AS d48:1**, Cer_EODS d57:0, Cer_NDS d18:0_23:0, Cer_NDS d21:0_24:0, Cer_NDS d24:0_24:0, Cer_NDS d26:0_24:0, Cer_NDS d43:0, **Cer_NDS d44:0**, Cer_NDS d47:0, Cer_NS d18:1_24:0, Cer_NS d18:1_24:1, EtherPC 18:0e_22:6, EtherPC 42:6e, EtherPE 18:0e_18:2, FA 18:2, FAHFA 18:1_18:0, FAHFA 20:4_20:3, HexCer_AP t18:0_23:0, **LPC 14:0/0:0**, LPC 20:4/0:0, **LPE 16:0**, PC 18:1_20:4, PC 19:0_22:6, PC 22:0_18:2, PE 16:0_20:4, PE 18:0_18:2, PE 19:0_18:2 | **Cer_ADS d47:0**, Cer_AP t22:0_24:0, **Cer_AS d48:1**, Cer_NDS d40:1, **Cer_NDS d44:0**, **LPC 14:0/0:0,** **LPE 16:0** |  |

Lipids altered in the EEF and EEM comparisons are indicated in boldface, lipids altered in the EEF and SEI comparisons are underlined, and lipids altered in the EEM and SEI comparisons are indicated in purple italics.

**Table S11.** Lipids with significant differential abundance in TLR4-KO mouse samples, separated by LFC.

| Positive ion mode **TLR4-KO mice** | EEF | EEM | SEI |
| --- | --- | --- | --- |
| LFC>0 | SM d31:1, TG 18:2_18:2_18:3 | LPC 0:0/18:0, LPC 0:0/18:2, LPC 19:0/0:0, LPC 20:1/0:0, LPC 20:3/0:0, LPC 22:1/0:0, LPC 22:5/0:0, PC 18:0_22:6, PC 34:1, PC 39:7, PC 40:10, PC 40:7, PC 42:5, SM d18:1_18:0, SM d32:2, SM d34:0, SM d35:1, SM d39:1, TG 52:7, TG 55:6, TG 57:7, TG 57:8 | TG 18:2_18:2_18:3, *TG 46:0* |
| LFC<0 | **TG 45:2** | DGTS 38:2, LPC 16:0/0:0, PC 18:1_20:4, TG 16:0_16:0_18:1, TG 16:0_16:1_16:1, TG 16:0_18:0_18:1, TG 16:0_18:1_18:1, **TG 45:2**, *TG 46:0*, TG 47:2, TG 48:1, TG 53:0, TG 54:0, TG 57:2 |  |

Lipids altered in the EEF and EEM comparisons are indicated in boldface, lipids altered in the EEF and SEI comparisons are underlined, and lipids altered in the EEM and SEI comparisons are indicated in purple italics.
